# The interplay between community and hospital *Enterococcus faecium* clones within health-care settings: a genomic analysis

**DOI:** 10.1016/S2666-5247(21)00236-6

**Published:** 2022-02

**Authors:** Sebastiaan J van Hal, Rob J L Willems, Theodore Gouliouris, Susan A Ballard, Teresa M Coque, Anette M Hammerum, Kristin Hegstad, Mette Pinholt, Benjamin P Howden, Surbhi Malhotra-Kumar, Guido Werner, Katsunori Yanagihara, Ashlee M Earl, Katherine E Raven, Jukka Corander, Rory Bowden, Enterococcal Study Group

**Affiliations:** a Department of Infectious Diseases and Microbiology, Royal Prince Alfred Hospital, Sydney, NSW, Australia; b Central Clinical School, University of Sydney, Sydney, NSW, Australia; c Department of Medical Microbiology, University Medical Center Utrecht, Utrecht, Netherlands; d Cambridge University Hospitals NHS Foundation Trust, Cambridge, UK; e Microbiological Diagnostic Unit Public Health Laboratory, The University of Melbourne at The Peter Doherty Institute for Infection and Immunity, Melbourne, VIC, Australia; f Department of Microbiology, Ramón y Cajal University Hospital and Ramón y Cajal Health Research Institute, Madrid, Spain; g Network Research Centre for Epidemiology and Public Health, Madrid, Spain; h Statens Serum Institut, Copenhagen, Denmark; i Norwegian National Advisory Unit on Detection of Antimicrobial Resistance, University Hospital of North-Norway, Department of Microbiology and Infection Control, Tromsø, Norway; j Department of Clinical Microbiology, Hvidovre University Hospital, Hvidovre, Denmark; k Laboratory of Medical Microbiology, Vaccine & Infectious Disease Institute, Universiteit Antwerpen, Wilrijk, Belgium; l National Reference Centre for Staphylococci and Enterococci, Division of Nosocomial Pathogens and Antibiotic Resistances, Department of Infectious Diseases, Robert Koch Institute, Wernigerode Branch, Wernigerode, Germany; m Department of Laboratory Medicine, Nagasaki University Graduate School of Biomedical Sciences, Nagasaki, Japan; n Infectious Disease & Microbiome Program, Broad Institute, Cambridge, MA, USA; o Department of Medicine, University of Cambridge, Cambridge, UK; p Department of Biostatistics, University of Oslo, Oslo, Norway; q Parasites and Microbes, Wellcome Sanger Institute, Saffron Walden, UK; r The Walter and Eliza Hall Institute of Medical Research, Parkville, VIC, Australia; s Department of Medical Biology, University of Melbourne, Melbourne, VIC, Australia; t Wellcome Centre for Human Genetics, University of Oxford, Oxford, UK

## Abstract

**Background:**

The genomic relationships among *Enterococcus faecium* isolates are the subject of ongoing research that seeks to clarify the origins of observed lineages and the extent of horizontal gene transfer between them, and to robustly identify links between genotypes and phenotypes. *E faecium* is considered to form distinct groups—A and B—corresponding to isolates derived from patients who were hospitalised (A) and isolates from humans in the community (B). The additional separation of A into the so-called clades A1 and A2 remains an area of uncertainty. We aimed to investigate the relationships between A1 and non-A1 groups and explore the potential role of non-A1 isolates in shaping the population structure of hospital *E faecium*.

**Methods:**

We collected short-read sequence data from invited groups that had previously published *E faecium* genome data. This hospital-based isolate collection could be separated into three groups (or clades, A1, A2, and B) by augmenting the study genomes with published sequences derived from human samples representing the previously defined genomic clusters. We performed phylogenetic analyses, by constructing maximum-likelihood phylogenetic trees, and identified historical recombination events. We assessed the pan-genome, did resistome analysis, and examined the genomic data to identify mobile genetic elements. Each genome underwent chromosome painting by use of ChromoPainter within FineSTRUCTURE software to assess ancestry and identify hybrid groups. We further assessed highly admixed regions to infer recombination directionality.

**Findings:**

We assembled a collection of 1095 hospital *E faecium* sequences from 34 countries, further augmented by 33 published sequences. 997 (88%) of 1128 genomes clustered as A1, 92 (8%) as A2, and 39 (4%) as B. We showed that A1 probably emerged as a clone from within A2 and that, because of ongoing gene flow, hospital isolates currently identified as A2 represent a genetic continuum between A1 and community *E faecium*. This interchange of genetic material between isolates from different groups results in the emergence of hybrid genomes between clusters. Of the 1128 genomes, 49 (4%) hybrid genomes were identified: 33 previously labelled as A2 and 16 previously labelled as A1. These interactions were fuelled by a directional pattern of recombination mediated by mobile genetic elements. By contrast, the contribution of B group genetic material to A1 was limited to a few small regions of the genome and appeared to be driven by genomic sweep events.

**Interpretation:**

A2 and B isolates coming into the hospital form an important reservoir for ongoing A1 adaptation, suggesting that effective long-term control of the effect of *E faecium* could benefit from strategies to reduce these genomic interactions, such as a focus on reducing the acquisition of hospital A1 strains by patients entering the hospital.

**Funding:**

Wellcome Trust.

## Introduction

*Enterococcus faecium* is a pathogen of global significance, causing serious hospital-associated infections. The impact of these infections is augmented by the high rates of acquired resistance, including to first-line antibiotics such as vancomycin.^1^, ^2^ The control of hospital-acquired *E faecium* infection is challenging because routes of transmission are often unclear, and the pathogen tends to persist in the hospital environment despite infection control campaigns that have successfully reduced the spread of other harmful nosocomial pathogens.

*E faecium* genomes are considered to form two distinct groups, A and B, corresponding to isolates originating from humans in a hospital setting (A) and those in a community setting (B).^3^, ^4^ Cluster A can be further split into two subgroups referred to as clades A1 and A2, on the basis of a detailed analysis in which A2 isolates were mostly collected from animals.^4^ Subsequent investigations of larger sample sets have confirmed the expansion of a tightly clustered group A (A1), comprising the majority of hospital isolates. However, these data did not support a single distinct A2 clade, but rather numerous A2 subgroups that appear to be largely host-specific, including a human A2 or non-A1 clade.^5^, ^6^, ^7^, ^8^ Controversy remains on whether A2 constitutes a single clade, paraphyletic with A1, or rather a series of earlier-branching lineages of the A group. Irrespective of this distinction, just as for group B, human A2 carriage is mostly seen in the gastrointestinal tract of individuals from the community, rather than those admitted to hospital. At the level of individuals, carriage is dynamic: strains are often observed to be replaced by the predominant hospital A1 clade on entering the health-care system or replaced by community strains upon leaving it.

**Research in context**

**Evidence before this study**

We searched PubMed for studies in English published between Jan 1, 1995, and Dec 31, 2019, using the terms “*Enterococcus faecium*”, and “sequencing” or “population structure”. We filtered the 124 and 76 studies identified including only those that used whole genome sequencing in datasets of more than 50 isolates. Several additional studies were identified by sifting through manuscript references. Following the initial description of *Enterococcus faecium* groupings A1, A2, and B, current studies have questioned the position or existence of A2 within this overall population structure.

**Added value of this study**

We showed that A1 emerged as a clone from within A2 and that, because of ongoing gene flow, hospital isolates currently identified as A2 represent a genetic continuum between A1 and community *E faecium*. By contrast, the contribution of B group genetic material to A1 is limited to a few small regions of the genome and appears to be driven by genomic sweep events. A2 isolates entering the hospital form an important reservoir for ongoing A1 adaptation, fuelled by a directional pattern of recombination mediated by mobile genetic elements.

**Implications of all the available evidence**

New hospital *E faecium* clones will continue to emerge, drawing genetic material—including antimicrobial resistance genes—from community clones entering the health-care system. Control of vancomycin-resistant *E faecium* in the longer term will thus remain a challenge, especially if infection control interventions are limited to stopping patient-to-patient transmission of strains that are already present in the hospital, rather than reducing opportunities for new and potentially adaptively favoured forms to arise, which can then acquire vancomycin resistance and present a direct threat.

Considering that *E faecium* is highly recombinogenic, genetic interactions between distinct *E faecium* groups might be important in the continuing evolution of hospital strains. However, this interaction remains largely undefined. In this study, we aimed to analyse a large global sample of hospital-isolated *E faecium* to resolve the interplay between A1 and non-A1 groups and explore the potential role of non-A1 isolates in shaping the population structure of hospital *E faecium*.

## Methods

### Isolate collection and data availability

We invited groups that had previously published *E faecium* genome data to contribute short-read sequence data. Participants were asked to select both vancomycin-susceptible and vancomycin-resistant *E faecium* sequence data from patients who were hospitalised and to ensure that isolates from the same hospital were not enriched by known outbreaks, on the basis of local epidemiological data.

### Classification of sequences

We trimmed short-read sequence data passing quality metrics using trimmomatic (version 0.38) before mapping against reference Aus0004 (GenBank CP003351, *vanB*-positive Australian, hospital, clade A1 isolate) using bwa (version 0.7.17-r1188).^9^, ^10^ We identified single nucleotide polymorphisms (SNPs) using FreeBayes (version 1.3.2-dirty) with variants considered present if the proportion of reads supporting the allele was greater than 90%.^11^ All SNPs were subsequently included, provided that more than 75% of isolates had data across that site. Insertions and deletions were excluded from the analysis.

We used informative SNPs to cluster isolates using the Hierarchical Bayesian Analysis of Population Structure (hierBAPs) software.^12^ We separated the hospital-based isolate collection into three groups, or so-called clades, known as A1, A2, and B, by augmenting the study genomes with 33 published sequences derived from human samples representing the previously defined genomic clusters clade B (n=8), A2 (n=11), and A1 (n=14).^4^ Subsequent clade assignment of study genomes was based on group comembership. In multiple runs of hierBAPS, groups A and B were recovered when the maximum level of clusters was set at two. All subsequent runs with increasing maximum level of clusters from two to 20 converged on the same posterior clustering estimate, in which group A split into two subgroups corresponding to the published A1 and A2 lineages. To exclude a possible misclassification error based on the species of origin, the analysis was repeated including an additional 19 non-human (animal and environmental) genomes, obtaining concordant results, in which human and non-human A2 isolates joined a single group.^4^

### Phylogenetic analysis and detection of recombination

We identified historic recombination events using ClonalFrameML (version 1.12) with an iterative approach.^2^, ^13^ Identified recombination events on each branch were masked before rebuilding the tree for phylogenetic reconstructions against the original dataset. We also assessed recombination using Gubbins (version 2.4.1), with all maximum likelihood phylogenetic trees constructed with a GTR+ϒ model implemented in RaxML (version 8.1.3).^14^, ^15^ Bootstrap analysis was implemented on the final phylogenetic trees with 100 independent maximum likelihood runs, with branch and node support inferred after 1000 bootstrap replicates.

### Pan-genome, resistome analysis, and identification of mobile genetic elements

We determined the pan-genome using contigs assembled with SPAdes, version 3.13.1, under the careful option.^16^ Contigs smaller than 2000 bp were removed and discarded from individual assemblies, which were annotated using prokka, version 1.13, before pan-genome discovery with Roary.^17^, ^18^

We identified the in-silico resistome using the de-novo assemblies, requiring at least 80% coverage and 95% sequence identity to a specific gene, using AMRFinder specifying *E faecium.*^15^

We examined the genomic data for mobile genetic elements (MGEs) using two approaches. The first identified the locations of chromosomal MGEs of the reference with use of the online tools ISFinder and RAST.^19^, ^20^ Mapped sequences were subsequently interrogated with a similar MGE considered present, provided that more than 85% coverage and more than 90% similarity was obtained across the query region. The second approach used the prokka annotations and Roary output to define MGEs within the assembled contigs.

All genome-wide analyses were undertaken on the same set of 10 kb windows relative to the reference sequence. Regions associated with the *vanB* transposon (2835430–2869240) were excluded.

### Chromosome painting and FineSTRUCTURE analyses

The ChromoPainter tool embedded within FineSTRUCTURE (version 2.0.3) was used to paint each genome, setting a starting mean recombination rate of 2·3 × 10^−7^.^21^ This method assigns every section of an isolate's genome as a succession of haplotype fragments originating from the closest matching sequence in the collection, or donor haplotypes with donor switches occurring at recombination breakpoints. Each painted genome is subsequently represented as a probability matrix of probable donors per 10 kb window. These painted genomes were aggregated by defined clades—A1, A2, and B—with similar admixture patterns across most of their genome representing a continuous line of ancestry. Hybrids were defined when the dominant proportion of painted loci arose from outside their assigned clade. Different admixture cutoffs were used to define a hybrid group, and a value higher than 30% was found to be the most informative when mapping back to the phylogeny and determining intergroup relationships.

### Recombination directionality

We further assessed highly admixed regions using fastGEAR, which identifies recombination events by looking for similar genomic segments between diverse clusters of data.^22^ Using a hidden Markov model, fastGEAR assigns the origin of DNA fragments relative to other sequences through identification of lineages. Recent recombination events do not affect all genomes, and thus directionality can be inferred. By contrast, so-called remote events reveal shared ancestry between genomes, consistent with segments arising from outside the sample set—for which the directionality of transfer cannot be inferred.

### Statistical analysis

We used the Kruskal-Wallis test for between-genomic group comparisons, with a p value lower than 0·05 considered significant. These comparisons were done with the R stats package, version 3.6.3.

### Role of the funding source

The funder of the study had no role in study design, data collection, data analysis, data interpretation, or writing of the report.

## Results

We assembled a collection of 1095 isolate sequences from 34 countries, from 1957 to 2016, of which 321 were newly sequenced and uploaded (PRJNA636894), with the remaining sequences (774) downloaded from the National Center for Biotechnology Information. An additional 52 sequences were downloaded and represented the previous genomic basis for *E faecium* groupings (appendix pp 3–45). Most isolates (770 [70%] of 1095) came from clinical infections, with the remainder split between colonisation (143 [13%]) and unknown (182 [17%]) sources.

With the Aus0004 genome as a reference to identify 261 543 variant positions across the 2·96 Mb genome, we grouped the genomes using hierBAPs.^12^ In terms of previously defined genomic groups, 997 (88%) of 1128 genomes clustered as A1, 92 (8%) as A2, and 39 (4%) as B. Despite all study genomes originating from patients who were hospitalised, previous well established associations would indicate that A2 and B genomes represent community *E faecium* isolates entering the hospital.^4^, ^7^

**Figure 1:**
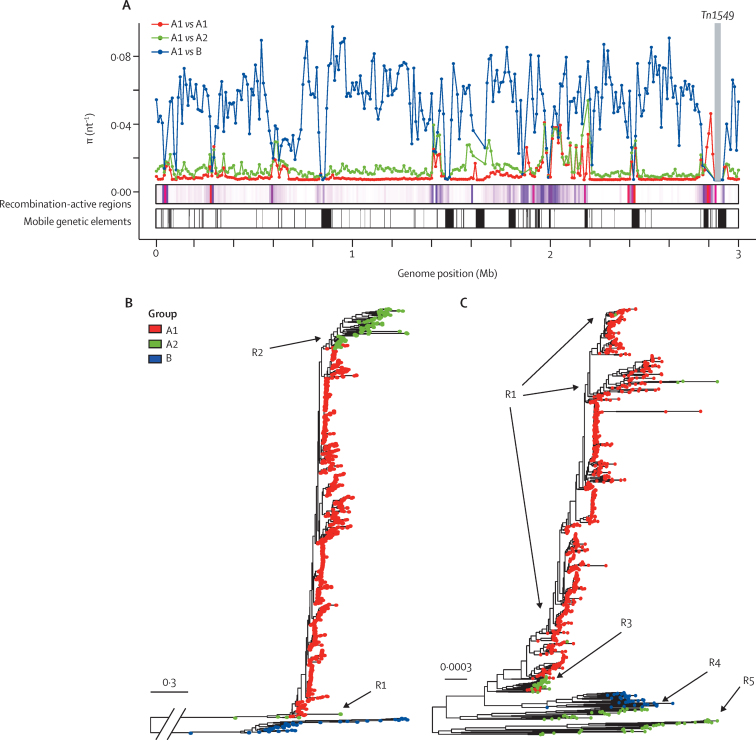
*Enterococcus faecium* population structure (A) Mean pairwise diversity (π, nt^−1^) within (A1 *vs* A1) and between (A1 *vs* A2 and A1 *vs* B) groups, plotted in 10 kb windows across the genome; *Tn1549*, carrying the *vanB* gene cluster, was excluded from the analysis and is depicted by the labelled grey box; regions where between-group diversity converges coincide in many instances with recombination-active regions and mobile genetic elements. (B, C) Maximum-likelihood phylogenies of 1128 *E faecium* genomes, before (B) and after (C) masking of recombination events identified using ClonalFrameML, with tips coloured by group assigned using Bayesian Analysis of Population Structure; subgroups of A2 isolates are labelled R1 to R5 according to their tree position; isolates labelled R2 in (B) split into subgroups R3, R4, and R5 in (C).

Plotting molecular diversity in 10 kb windows along the genome supported the distinct identities of the 3 groups, with mean between-group diversity approximately five times (A1 *vs* A2) and 40 times (A1 *vs* B) that within A1, across the majority of the genome, whereas the mean diversity was 28 times in A2 versus B. However, in several regions of the genome, within-group and between-group diversity converged, corresponding to regions of recombination, coinciding with and mediated by MGEs (figure 1A).

To investigate relationships between the three groups, we constructed a maximum-likelihood phylogeny, which confirmed the deep split between groups A and B. A2 isolates separated into two subgroups, labelled R1 and R2, occupying distinct branches from A1 (figure 1B).

We observed a relative rate of recombination to mutation greater than 4 across the genome, consistent with high rates of recombination (appendix p 2).^23^ Irrespective of method used (Gubbins *vs* ClonalFrameML), we identified more historic recombination events in comparisons of B with A1 genomes than in comparisons of A1 with A2 genomes. The masking of identified recombination events excluded on average 2·5 Mb, or 85% of B genomes, distorting the inferred relationships between B and A genomes. Because of the shorter time depth, this effect was less marked when comparing A1 with A2 isolates alone (with an average of 0·9 Mb or 33% of genome masked as a result of recombination), with the R2 subgroup split into distinct subgroups (ancestral to A1) and labelled R3, R4, and R5 (figure 1C).

A2 hospital *E faecium* genomes were identified in 19 (56%) of 34 sampled countries, indicating that observations about the distribution of A2 were not driven by a single region of the world. Similarly, hospital B isolates were not restricted to any one country.

**Figure 2:**
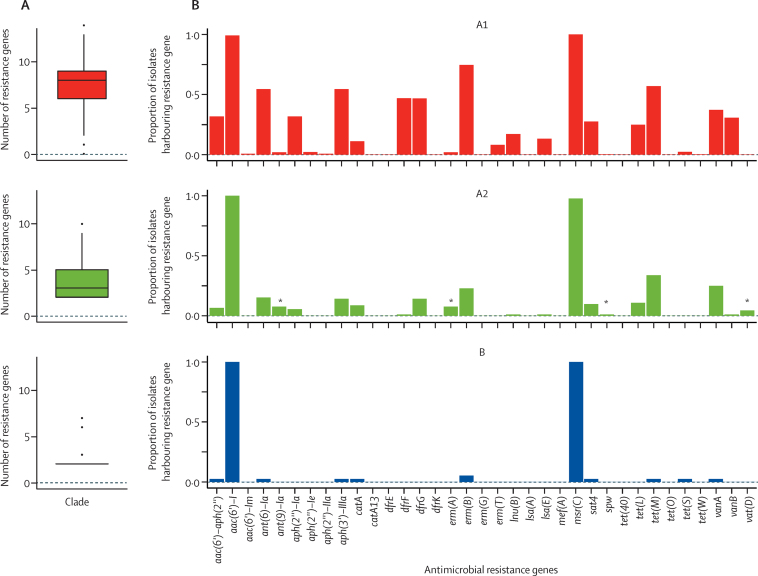
Antimicrobial resistance (A) Box and whisker plot of the number of resistance genes carried by each of the *E faecium* clades A1, A2, and B. (B) The proportion of isolates from each clade harbouring any specific resistance gene (labelled on the x-axis). *Indicates a gene that is preferentially carried by A2 clade compared with A1.

A pan-genome analysis identified 1854 core genes (each found in more than 95% of all isolates) from a total of 18 468 detected genes. Several genes (299 in B and 23 in A2) were restricted (found in fewer than 5% of A1 isolates and more than 50% of either A2 or B) to non-A1 isolates (appendix pp 46–57). Most of such genes were annotated as hypothetical proteins. The remaining genes were found only in B isolates and included genes associated with carbohydrate metabolism, phosphotransferase systems, and iron transporters, recapitulating previous associations.^4^ Notably, genomes belonging to A2 and B contained genes associated with MGEs not found in A1 isolates. Non-A1 isolates harboured significantly fewer resistance genes (p<0·001) than hospital isolates; all isolates harboured an aminoglycoside resistance (*aac(6)-I*) gene and an efflux pump (*msrC*) encoding macrolide resistance. We observed an incremental increase in the median number of resistance genes, from two in B and three in A2, to eight in A1 (figure 2A). Vancomycin resistance was seen in 19 (21%) of A2 isolates and in one (3%) of 39 B isolates, consistent with gene movement from A1 to non-A1 isolates from patients entering the health-care system. Nevertheless, non-A1 isolates were a small but important contribution to the overall hospital antimicrobial resistance reservoir with the detection of *qacC*, encoding quaternary ammonium compound resistance, found only in two A2 genomes, and several other resistance genes preferentially carried by A2 genomes (figure 2B).

**Figure 3:**
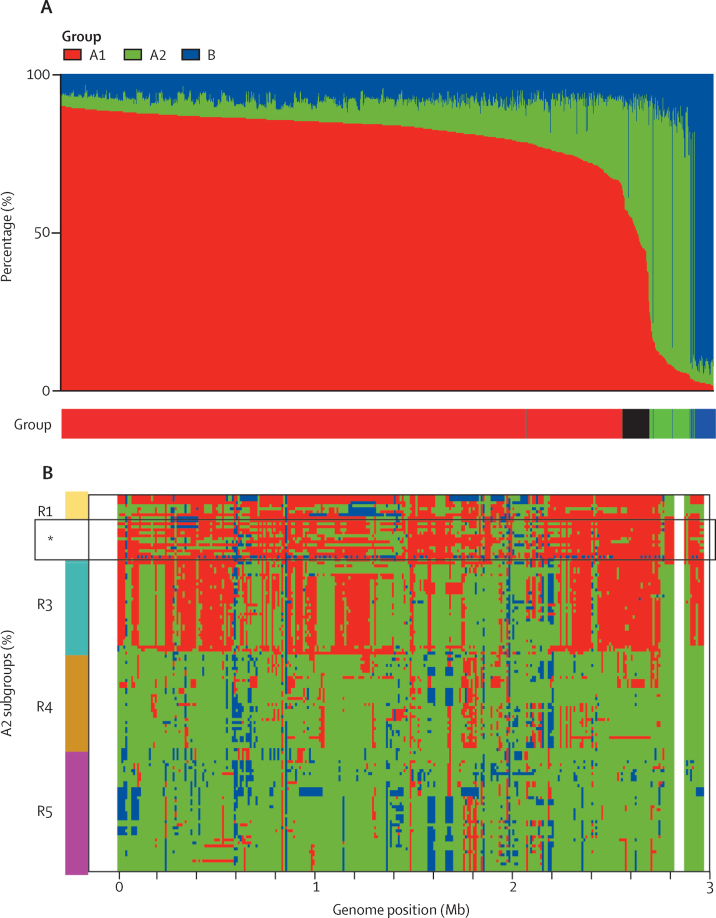
Chromosome painting assessing admixture in *Enterococcus faecium* (A) Total ancestry contribution from each of the A1, A2, and B groups by isolate, arranged in decreasing order of A1 ancestry; the legend beneath shows Bayesian Analysis of Population Structure group assignment, with hybrids denoted in black. (B) Chromosome painting of fragments along the genome for A2 isolates arranged by position on phylogeny (figure 1C), with subgroups shown by the coloured bar; R1 subgroup includes isolates assigned to A2 that have little estimated A2 ancestry; R4 and R5 are considered pure A2 isolates, whereas isolates within the R3 subgroup represent a probable clonal hybrid between A1 and A2. *This panel contains A1-labelled hybrid isolates falling within R3 on the phylogeny, which share a pattern of co-ancestry, distinct from other R3 isolates.

Using ChromoPainter, we painted the 1128 genomes, clustering isolates by their hierBAPs assignment within the same 10 kb windows.^21^ Across most of the genome, the A1, A2, and B groups maintained their own identities despite significant recombination that can shuffle phylogenetic relationships. However, within our collection, we found a small proportion of isolates (49 [4·3%] of 1128) in which more than 30% of the genomic content could be traced to an alternative group. The 33 previously A2-labelled and 15 A1-labelled isolates (48 [4·3%] of 1128 sampled isolates) represent hybrid genomes that reflect a continuum of ancestry between A1 and A2 (figure 3A). We detected a single A1-labelled hybrid with B, suggesting that a similar continuum exists between A1 and B. We found no A2–B hybrids in our hospital-derived dataset, possibly reflecting the relatively reduced opportunities for these groups to interact within the hospital.

We noted no distinctive clinical or genomic characteristics among the 49 hybrid isolates, of which 31 (63%) represented infection isolates and ten (21%) harboured the vancomycin-resistance gene cluster.

Patterns of admixture between groups also drive overall phylogenetic relationships, with the position of A2 subgroups completely explained by their distinct genomic compositions and differing contributions of A1 ancestry. The R1 and R3 phylogenetic subgroups comprised hybrids with varying estimated proportions of A1 and A2 ancestry, whereas R4 and R5 accounted for non-hybrid isolates representing some of the diversity of the A2 group (figure 3B). By contrast with R1 isolates, the tight grouping of R3 genomes on the phylogeny suggests a single hybridisation event followed by clonal expansion. Accordingly, the A1 isolates that sit phylogenetically within the R3 genomes (figure 3B) are A1-labelled hybrids with distinct co-ancestry patterns from A2-labelled R3 isolates, across a minority of the genome.

Chromosome painting also revealed subsequent diversification of hybrid clones through the import of genetic material, including material representing group B sequences (figure 3B). These patterns confirmed that large-scale recombination was responsible for establishing distinct new genetic identities in A1 *E faecium*. Together with the presumption that A1 isolates primarily circulate in hospitals, A1–A2 hybrids continue to arise within and form part of the hospital-resident microbiota, and thus provide potential raw material for future forms of hospital *E faecium*, distinct from the more transient (unrecombined) A2 and B lineages.

**Figure 4:**
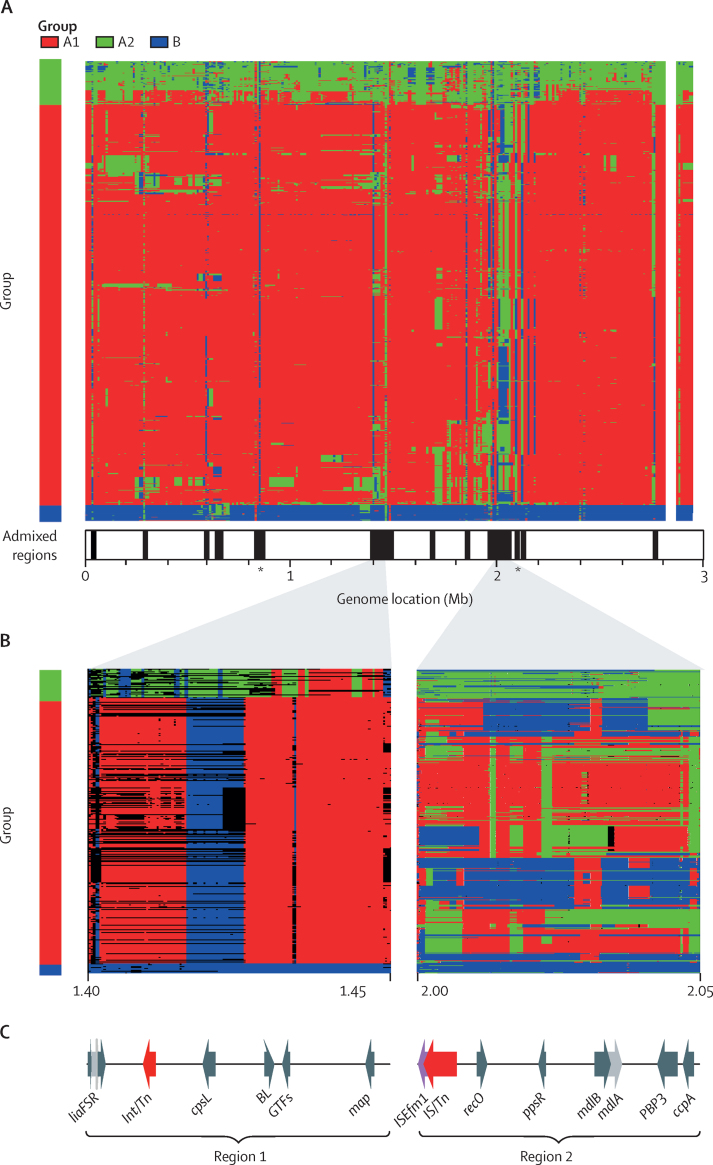
Extensive admixture and directionality of recombination in *Enterococcus faecium* (A) Chromosome painting along the genome for all 1128 isolates (rows), presented by group in order of the recombination-masked tree (figure 1C) and coloured by the inferred group of origin of each segment; legend indicates the Bayesian Analysis of Population Structure group assignment with the same colour key; highly admixed regions (more than 75% of isolates in any one group containing material from another group) are shown by the black lines in the panel underneath by genome position; *location of the *amidase* (left) and *lgt* (right) genes. (B) Recombination events inferred by fastGEAR of two representative 50 kb segments with the origin and the directionality of the sequences inferred by group colour within the panels; both remote (coloured by group) and recent (depicted by black lines) recombination events are shown within panel corresponding to region 1; only recent (coloured by group) events are depicted in the second panel corresponding to region 2. (C) Annotations of genes of interest are shown across the two regions; only genes with a known function in *E faecium* and those associated with mobile genetic elements are shown; region 1 genes are the *LiaFSR* gene cluster, Int/Tn, *cpsL*, BL, GTFs, and *map*; region 2 genes are *ISEfm1*, IS/Tn, *recO, ppsR, mdlA* and *mdlB, PBP3*, and *ccpA.*

Several short segments of the genome contained sequences from a different group, including several regions of high admixture (more than 75% of isolates in any one group contained imported sequences from another group; figure 4A). These regions were found in all groups, corresponding to genomic segments of converging diversity and were associated with MGEs (figure 1A).

Using fastGEAR, we examined the direction of horizontal transfer in two representative highly admixed regions (figure 4B).^22^ Focusing on so-called remote events (those that are mapped back to the root of the tree) in region 1, a single haplotype was observed across all hospital isolates. Genes of interest in this region included the *liaS, liaF*, and *liaR* gene cluster that forms part of a three-component regulatory system involved in cell envelope stress responses, and the polysaccharide biosynthesis protein (CpsL) associated with virulence in streptococcal species (figure 4C). Within A2 isolates, we observed a distinct pattern of co-ancestry with group B in region 1 and remote events appearing to originate from A1, including a class C b-lactamase that has been associated with ampicillin resistance in *E faecium*.^24^

Focusing on recent events in region 2, we observed several distinct patterns of transfer from A2 and B into A1. Despite the number of probable events observed and their association with MGEs, genomic diversity within groups remained relatively low across this region. Evidence of restriction and dissemination of a single haplotype was seen in the *ccpA* gene (encoding the catabolite control protein A), a crucial regulator of carbon metabolism in Gram-positive bacteria.

Exploring the other highly admixed regions revealed convergent evolution leading to a single genomic form. Two genes of interest were noted. One, *lgt* (located at 2·06 Mb), encoding a prolipoprotein diacylglyceryl transferase, plays a crucial role in membrane anchoring, growth, virulence, and stress responses, especially under oxidative stress.^25^ A second gene with a similar pattern of variation, the amidase gene (located at 0·85 Mb) encodes the N-acetylmuramoyl-L-alanine amidase enzyme, involved in biosynthesis of peptidoglycan integral to *E faecium* cell walls.^26^

## Discussion

In this study, we contribute to resolving the uncertainties about the *E faecium* global population structure by assembling the largest-to-date collection of genomes from patients who were hospitalised, using a sampling strategy designed to maximise the representation of both colonising and infection isolates. Benefiting from both the broad-based sample set and an appropriate analysis framework, our data supports the distinctiveness of separate A and B groups.

Additionally, we showed that group A isolates do split into two distinguishable subgroups, approximately equivalent to previous A1 and A2 clades. Some of the previous controversy about the status of A2 is thus probably explained by sampling strategies that might have left the small hospital A2 pool unsampled. After masking of identified recombination events, A2 isolates could be distinguished into subgroups seemingly ancestral to the lower-diversity A1 subgroup. Moreover, our observations suggest that A1 initially emerged as a clone from A2, evidenced by A1's low divergence from A2 compared with B. This emergence probably occurred in the hospital, an environment in which high rates of recombination continue to occur. These events are facilitated by MGEs within and between groups, as evidenced by regions of sequence convergence across *E faecium* populations.

Subgroup relationships were examined in more detail by use of a co-ancestry approach. The advantage of this approach is that identified relationships are not dependent on incomplete assessments of recombination through phylogenetic reconstructions and subsequent exclusion of substantial amounts of genomic data leading to loss of inferences as seen with B genomes. By contrast, chromosome painting provides a coherent view of genetic exchange and showed a continuum of co-ancestry patterns linking groups because of the interplay between the different *E faecium* groups found in hospitals. New genomic forms arise from this interplay, presumably within the gastrointestinal tracts of patients who are hospitalised. These forms have the potential to become established as either novel A1 sublineages, with further differentiation through recurrent recombination (as we have showed in other work)^27^ or as more distinct new lineages that would parallel the origins of A1.

Together these data emphasise the genomic distinctions between the three groups and the high rates of recombination between resident hospital-adapted A1 isolates and enterococcal A2 or B (ie, community) isolates entering the hospital. The observed direction of exchange in sampled isolates is preferentially from A2 or B to A1, and it probably includes some ascertainment bias (a limitation of our study of hospital isolates). Nonetheless we showed the flux of genetic variation into the hospital, enhancing opportunities to form new lineages by recombination with already-adapted lineages. Therefore, the population structure of A1 continues to evolve by introgression of genes and through the formation of hybrid genomes. Higher rates of introduction and mixing of genomically diverse lineages can only make the formation of new, more highly adapted and potentially difficult to control clones of *E faecium* more likely.

Strictly, our analysis applies only to *E faecium* obtained from humans who were hospitalised. It remains unknown whether our findings reflect *E faecium* interactions between isolates sampled from other settings, such as environmental or animal sources. Nevertheless, it is clear (from other work) that A2-like isolates are seen in animals and the environment, and similar genomes are detected in humans and companion animals, thus the rate of genomic interchange is likely to reflect opportunities for interaction.^7^

The best terminology to describe A1's relationship to the other diverse A subgroups is likely to remain uncertain until further research confirms the current findings, establishes the full set of such groups in different environments and hosts, and perhaps revisits the taxonomic relationship between the A and B groups of *E faecium*.

Most of the recombination in this study appears to affect a minority of the genome, corresponding to highly admixed regions and so-called remote events, in which sequences have been imported from outside our sampled *E faecium* collection. Although the overall effect of these regions on A1 adaptation remains unknown, such segments tend to be within previously documented hot-spots for further recombination.^2^ One such region, the *LiaFSR* gene cluster associated with stress responses, carries among A1 isolates a limited range of alleles apparently originating through remote recombination events from B genomes and is suggestive of directional selection with subsequent clonal expansion implying possible selective sweep events. Subsequent imports of A2-like sequences appear to have supplied a distinct new haplotype, the only one on which daptomycin resistance has been documented.^27^ Therefore, although remote recombination seems crucial to the genomic diversity of this gene cluster, group A2 clearly represents the more important recent and ongoing source of genetic material for A1.^28^ Exploring the other highly admixed regions (carrying the *lgt* and *amidase* genes) revealed patterns of convergent evolution leading to a single genomic form suggestive of inter-lineage recombination in the face of selective pressure.

MGEs play a crucial role in recombination in *E faecium*, with allele replacement events across A1, generally associated with genes involved in environmental adaptation and stress responses.^7^ Although the adaptive consequences of such genomic sweeps remain unclear, it is tantalising to speculate that these might be the events that explain the establishment and expansion of A1 in the health-care sector.

Antimicrobial resistance does not feature heavily in our analysis, despite the association of many resistance phenotypes in *E faecium* mediated by MGEs. Possible reasons include the relatively small non-A1 resistance gene pool and the dominance of A1 isolates within the hospital, such that resistance mobilisation most likely represents within-group recombination. This characteristic of resistance dissemination separate from genome adaptation might explain why, as others have observed, antimicrobial resistance remains disconnected from A1 clonal success and expansion.^6^, ^29^

Our study is limited by the small number of genomes originating from several known high burden *E faecium* countries such as the USA. Sparse sampling from Asia and Africa might also have influenced our conclusions.

In conclusion, we provide a detailed analysis based on the patterns of co-ancestry of segments of the *E faecium* genome using chromosome painting. This has allowed us to elucidate key aspects of the population structure and dynamics of hospital-associated *E faecium* on a global scale, resolving long-standing questions about the relationships and interplay between *E faecium* genomes.

## Data sharing

All new short-read sequence data (n=321) was uploaded to the National Center for Biotechnology Information (NCBI) under project number PRJNA63689. The remaining sequences (n=807) were downloaded from NCBI with project numbers and associated metadata provided in the appendix (pp 3–45).

## Declaration of interests

We declare no competing interests.
