# Supplementary material for: The interplay between community and hospital Enterococcus faecium clones within health-care settings: a genomic analysis
Source: Lancet Microbe. 2022 Feb;3(2):e133–41. doi: 10.1016/S2666-5247(21)00236-6 (PMC8810393; doi:10.1016/S2666-5247(21)00236-6)
Supplement: Supplementary appendix [file mmc1.pdf]

THE LANCET
Microbe

Supplementary appendix

This appendix formed part of the original submission and has been peer reviewed. We post it as supplied by the authors.

Supplement to: van Hal SJ, Willems RJL, Gouliouri T, et al. The interplay between community and hospital *Enterococcus faecium* clones within health-care settings: a genomic analysis. *Lancet Microbe* 2022; published online Jan 18. https://doi.org/10.1016/S2666-5247(21)00236-6.

Table of Contents

***Supplementary Figure 1: Recombination across E. faecium isolates.* 2**

***Supplementary Table 1: Isolate details* 3**

***Supplementary Table 2: Gene proportions by hBAPs group* 46**

Supplementary Figure 1: Recombination across *E. faecium* isolates.

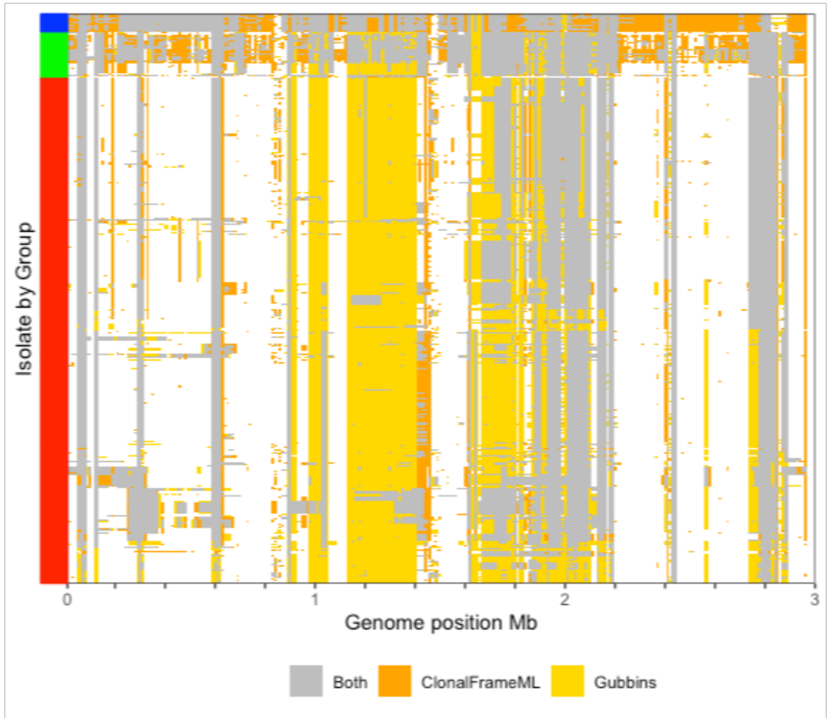

Recombination tracts identified using clonalFrameML and Gubbins across the genome for isolates grouped by hBAPs group. Grey depicts regions that were detected by both methods with orange and yellow tracts indicating regions detected by clonalFrameML and Gubbins alone respectively.

Supplementary Table 1: Isolate details

Study No.	BioProject	BioSample	Year[1]	Country[2]	Origin[3]	Gene[4]	hBAPs[5]	Host[6]
E0001	PRJNA636894	SAMN15088033	1997	Spain	Infection	None	B	Human
E0002	PRJNA636894	SAMN15088034	2009	Spain	Screening	None	B	Human
E0003	PRJNA636894	SAMN15088035	2009	Spain	Screening	None	B	Human
E0004	PRJNA636894	SAMN15088036	1997	Spain	Infection	None	B	Human
E0005	PRJNA636894	SAMN15088037	2009	Spain	Screening	None	B	Human
E0006	PRJNA636894	SAMN15088038	2009	Spain	Screening	None	B	Human
E0007	PRJNA636894	SAMN15088039	2009	Spain	Screening	None	B	Human
E0008	PRJNA636894	SAMN15088040	2011	Spain	Infection	None	A1	Human
E0009	PRJNA636894	SAMN15088041	2004	Spain	Infection	None	A1	Human
E0010	PRJNA636894	SAMN15088042	2012	Spain	Infection	None	A1	Human
E0011	PRJNA636894	SAMN15088043	2012	Spain	Infection	None	A1	Human
E0012	PRJNA636894	SAMN15088044	2006	Spain	Infection	None	A1	Human
E0013	PRJNA636894	SAMN15088045	2008	Spain	Infection	None	A1	Human
E0014	PRJNA636894	SAMN15088046	2009	Spain	Infection	None	A1	Human
E0015	PRJNA636894	SAMN15088047	2011	Spain	Screening	None	A2	Human
E0016	PRJNA636894	SAMN15088048	2010	Spain	Screening	None	A2	Human
E0017	PRJNA636894	SAMN15088049	2006	Spain	Infection	None	A2	Human
E0018	PRJNA636894	SAMN15088050	2010	Spain	Infection	None	A1	Human
E0019	PRJNA636894	SAMN15088051	2010	Spain	Infection	None	A1	Human
E0020	PRJNA636894	SAMN15088052	2011	Spain	Infection	None	A1	Human
E0021	PRJNA636894	SAMN15088053	2009	Spain	Screening	None	A2	Human
E0022	PRJNA636894	SAMN15088054	2009	Spain	Screening	None	A2	Human
E0023	PRJNA636894	SAMN15088055	2008	Spain	Infection	None	A2	Human

E0024	PRJNA636894	SAMN15088056	2009	Spain	Screening	None	A2	Human
E0025	PRJNA636894	SAMN15088057	2010	Spain	Screening	None	A2	Human
E0026	PRJNA636894	SAMN15088058	1999	Spain	Infection	None	A2	Human
E0027	PRJNA636894	SAMN15088059	2010	Spain	Infection	None	A1	Human
E0028	PRJNA636894	SAMN15088060	2010	Spain	Infection	None	A1	Human
E0029	PRJNA636894	SAMN15088061	2011	Spain	Infection	None	A1	Human
E0030	PRJNA636894	SAMN15088062	2010	Spain	Infection	None	A1	Human
E0031	PRJNA636894	SAMN15088063	2011	Spain	Infection	vanB	A1	Human
E0032	PRJNA636894	SAMN15088064	2009	Spain	Screening	None	A1	Human
E0033	PRJNA636894	SAMN15088065	2010	Spain	Infection	None	A2	Human
E0034	PRJNA636894	SAMN15088066	2011	Spain	Infection	None	A2	Human
E0035	PRJNA636894	SAMN15088067	1995	Spain	Infection	None	A2	Human
E0036	PRJNA636894	SAMN15088068	1995	Spain	Infection	None	A2	Human
E0037	PRJNA636894	SAMN15088069	2009	Spain	Screening	None	A1	Human
E0038	PRJNA636894	SAMN15088070	2002	Portugal	Infection	vanA	A1	Human
E0039	PRJNA636894	SAMN15088071	2004	Singapore	Unknown	vanB	A1	Human
E0040	PRJNA636894	SAMN15088072	1997	Australia	Infection	vanA	A1	Human
E0041	PRJNA636894	SAMN15088073	2000	Argentina	Screening	vanA	A1	Human
E0042	PRJNA636894	SAMN15088074	1992	USA	Unknown	vanA	A1	Human
E0043	PRJNA636894	SAMN15088075	1997	Argentina	Screening	vanA	A1	Human
E0044	PRJNA636894	SAMN15088076	2009	Spain	Infection	vanA	A1	Human
E0045	PRJNA636894	SAMN15088077	1996	Finland	Unknown	vanA	A1	Human
E0046	PRJNA636894	SAMN15088078	2003	Spain	Infection	vanA	A1	Human
E0047	PRJNA636894	SAMN15088079	2000	Saudi Arabia	Unknown	vanA	A1	Human
E0048	PRJNA636894	SAMN15088080	2003	Tunisia	Unknown	vanA	A1	Human
E0049	PRJNA636894	SAMN15088081	Unknown	Brazil	Unknown	vanA	A1	Human
E0050	PRJNA636894	SAMN15088082	1991	USA	Unknown	vanB	A1	Human

E0051	PRJNA636894	SAMN15088083	2004	Spain	Infection	vanB	A1	Human
E0052	PRJNA636894	SAMN15088084	2005	Spain	Infection	vanB	A1	Human
E0053	PRJNA636894	SAMN15088085	2006	Spain	Infection	vanB	A1	Human
E0054	PRJNA636894	SAMN15088086	2004	Spain	Unknown	vanB	A1	Human
E0055	PRJNA636894	SAMN15088087	2005	Poland	Infection	vanA	A1	Human
E0056	PRJNA636894	SAMN15088088	2006	Germany	Infection	vanA	A1	Human
E0057	PRJNA636894	SAMN15088089	2002	Italy	Infection	vanB	A1	Human
E0058	PRJNA636894	SAMN15088090	2000	Saudi Arabia	Unknown	vanA	A1	Human
E0059	PRJNA636894	SAMN15088091	Unknown	Portugal	Unknown	vanA	A1	Human
E0060	PRJNA636894	SAMN15088092	2005	Denmark	Unknown	vanA	A1	Human
E0061	PRJNA636894	SAMN15088093	2005	Poland	Screening	vanA	A1	Human
E0062	PRJNA636894	SAMN15088094	2003	Chile	Infection	vanB	A1	Human
E0063	PRJNA636894	SAMN15088095	2004	Chile	Infection	vanB	A1	Human
E0064	PRJNA636894	SAMN15088096	1999	Portugal	Infection	vanA	A1	Human
E0065	PRJNA636894	SAMN15088097	2002	Portugal	Infection	vanA	A1	Human
E0066	PRJNA636894	SAMN15088098	2001	Australia	Unknown	vanB	A1	Human
E0067	PRJNA636894	SAMN15088099	1998	Argentina	Screening	vanA	A1	Human
E0068	PRJNA636894	SAMN15088100	2001	Italy	Infection	vanA	A1	Human
E0069	PRJNA636894	SAMN15088101	2005	Spain	Screening	vanA	A1	Human
E0070	PRJNA636894	SAMN15088102	2006	Spain	Unknown	vanA	A1	Human
E0071	PRJNA636894	SAMN15088103	2005	Paraguay	Unknown	vanA	A1	Human
E0072	PRJNA636894	SAMN15088104	1998	Germany	Infection	vanA	A1	Human
E0073	PRJNA636894	SAMN15088105	2008	Spain	Infection	vanA	A1	Human
E0074	PRJNA636894	SAMN15088106	2004	Germany	Infection	vanA	A1	Human
E0075	PRJNA636894	SAMN15088107	2004	USA	Unknown	vanA	A1	Human
E0076	PRJNA636894	SAMN15088108	2005	Germany	Infection	vanA	A1	Human
E0077	PRJNA636894	SAMN15088109	2005	Denmark	Unknown	vanB	A1	Human

E0078	PRJNA636894	SAMN15088110	2000	Saudi Arabia	Unknown	vanA	A2	Human
E0079	PRJNA636894	SAMN15088111	2005	Canada	Unknown	vanA	A1	Human
E0080	PRJNA636894	SAMN15088112	2006	Paraguay	Unknown	vanA	A1	Human
E0081	PRJNA636894	SAMN15088113	2002	Portugal	Infection	vanA	A2	Human
E0082	PRJNA636894	SAMN15088114	Unknown	Brazil	Unknown	vanA	A1	Human
E0083	PRJNA636894	SAMN15088115	1987	France	Screening	vanA	A2	Human
E0084	PRJNA636894	SAMN15088116	1998	Spain	Infection	vanB	A1	Human
E0085	PRJNA636894	SAMN15088117	1996	Finland	Unknown	vanB	A1	Human
E0086	PRJNA636894	SAMN15088118	2002	Spain	Infection	vanB	A1	Human
E0087	PRJNA636894	SAMN15088119	2005	Spain	Infection	vanA	A1	Human
E0088	PRJNA636894	SAMN15088120	Unknown	Spain	Unknown	vanA	A1	Human
E0089	PRJNA636894	SAMN15088121	2005	Spain	Unknown	vanA	A1	Human
E0090	PRJNA636894	SAMN15088122	2005	Spain	Unknown	vanA	A1	Human
E0091	PRJNA636894	SAMN15088123	2009	Spain	Infection	vanA	A1	Human
E0092	PRJNA636894	SAMN15088124	2003	Spain	Infection	vanB	A1	Human
E0093	PRJNA636894	SAMN15088125	2004	Spain	Infection	vanB	A1	Human
E0094	PRJNA636894	SAMN15088126	1999	Spain	Infection	vanA	A1	Human
E0095	PRJNA636894	SAMN15088127	2005	Spain	Unknown	vanA	A1	Human
E0096	PRJNA336433	SAMN05510547	2015	Australia	Unknown	vanA	A1	Human
E0097	PRJNA336433	SAMN05510548	2015	Australia	Unknown	vanA	A1	Human
E0098	PRJNA336433	SAMN05510549	2015	Australia	Unknown	vanB	A1	Human
E0099	PRJNA336433	SAMN05510550	2015	Australia	Unknown	vanB	A1	Human
E0100	PRJNA336433	SAMN05510551	2015	Australia	Unknown	vanA	A1	Human
E0101	PRJNA336433	SAMN05510552	2015	Australia	Unknown	vanB	A1	Human
E0102	PRJNA336433	SAMN05510553	2015	Australia	Unknown	vanB	A1	Human
E0103	PRJNA336433	SAMN05510554	2015	Australia	Unknown	vanB	A1	Human
E0104	PRJNA336433	SAMN05510555	2015	Australia	Unknown	vanB	A1	Human

E0105	PRJNA336433	SAMN05510556	2015	Australia	Unknown	vanA	A1	Human
E0106	PRJNA336433	SAMN05510557	2015	Australia	Unknown	vanB	A1	Human
E0107	PRJNA336433	SAMN05510558	2015	Australia	Unknown	vanA	A1	Human
E0108	PRJNA336433	SAMN05510559	2015	Australia	Unknown	vanB	A1	Human
E0109	PRJNA336433	SAMN05510560	2015	Australia	Unknown	vanB	A1	Human
E0110	PRJNA336433	SAMN05510561	2015	Australia	Unknown	vanA	A1	Human
E0111	PRJNA336433	SAMN05510562	2015	Australia	Unknown	vanA	A1	Human
E0112	PRJNA336433	SAMN05510563	2015	Australia	Unknown	vanB	A1	Human
E0113	PRJNA336433	SAMN05510564	2015	Australia	Unknown	vanB	A1	Human
E0114	PRJNA336433	SAMN05510565	2015	Australia	Unknown	vanB	A1	Human
E0115	PRJNA336433	SAMN05510566	2015	Australia	Unknown	vanB	A1	Human
E0116	PRJNA336433	SAMN05510567	2015	Australia	Unknown	vanB	A1	Human
E0117	PRJNA336433	SAMN05510568	2015	Australia	Unknown	vanB	A1	Human
E0118	PRJNA336433	SAMN05510569	2015	Australia	Unknown	vanB	A1	Human
E0119	PRJNA336433	SAMN05510570	2015	Australia	Unknown	vanB	A1	Human
E0120	PRJNA336433	SAMN05510571	2015	Australia	Unknown	vanB	A1	Human
E0121	PRJNA336433	SAMN05510572	2015	Australia	Unknown	vanB	A1	Human
E0122	PRJNA336433	SAMN05510573	2015	Australia	Unknown	vanA	A1	Human
E0123	PRJNA336433	SAMN05510574	2015	Australia	Unknown	vanB	A1	Human
E0124	PRJNA336433	SAMN05510575	2015	Australia	Unknown	vanB	A1	Human
E0125	PRJNA336433	SAMN05510576	2015	Australia	Unknown	vanB	A1	Human
E0126	PRJNA336433	SAMN05510577	2015	Australia	Unknown	vanB	A1	Human
E0127	PRJNA336433	SAMN05510578	2015	Australia	Unknown	vanA	A1	Human
E0128	PRJNA336433	SAMN05510579	2015	Australia	Unknown	vanB	A1	Human
E0129	PRJNA336433	SAMN05510580	2015	Australia	Unknown	vanB	A1	Human
E0130	PRJNA336433	SAMN05510581	2015	Australia	Unknown	vanB	A1	Human
E0131	PRJNA336433	SAMN05510582	2015	Australia	Unknown	vanB	A1	Human

E0132	PRJNA336433	SAMN05510583	2015	Australia	Unknown	vanA	A1	Human
E0133	PRJNA336433	SAMN05510584	2015	Australia	Unknown	vanA	A1	Human
E0134	PRJNA336433	SAMN05510585	2015	Australia	Unknown	vanB	A1	Human
E0135	PRJNA336433	SAMN05510586	2015	Australia	Unknown	vanB	A1	Human
E0136	PRJNA336433	SAMN05510587	2015	Australia	Unknown	vanB	A1	Human
E0137	PRJNA336433	SAMN05510588	2015	Australia	Unknown	vanA	A1	Human
E0138	PRJNA336433	SAMN05510589	2015	Australia	Unknown	vanB	A1	Human
E0139	PRJNA336433	SAMN05510590	2015	Australia	Unknown	vanB	A1	Human
E0140	PRJNA336433	SAMN05510591	2015	Australia	Unknown	vanB	A1	Human
E0141	PRJNA336433	SAMN05510592	2015	Australia	Unknown	vanA	A1	Human
E0142	PRJNA336433	SAMN05510593	2015	Australia	Unknown	vanA	A1	Human
E0143	PRJNA336433	SAMN05510594	2015	Australia	Unknown	vanB	A1	Human
E0144	PRJNA336433	SAMN05510595	2015	Australia	Unknown	vanB	A1	Human
E0145	PRJNA336433	SAMN05510596	2015	Australia	Unknown	vanB	A1	Human
E0146	PRJNA336433	SAMN05510597	2015	Australia	Unknown	vanB	A1	Human
E0147	PRJNA336433	SAMN05510598	2015	Australia	Unknown	vanB	A1	Human
E0148	PRJNA336433	SAMN05510599	2015	Australia	Unknown	vanA	A1	Human
E0149	PRJNA336433	SAMN05510600	2015	Australia	Unknown	vanB	A1	Human
E0150	PRJNA336433	SAMN05510601	2015	Australia	Unknown	vanB	A1	Human
E0151	PRJNA336433	SAMN05510602	2015	Australia	Unknown	vanB	A1	Human
E0152	PRJNA336433	SAMN05510603	2015	Australia	Unknown	vanA	A1	Human
E0153	PRJNA336433	SAMN05510604	2015	Australia	Unknown	vanB	A1	Human
E0154	PRJNA336433	SAMN05510605	2015	Australia	Unknown	vanB	A1	Human
E0155	PRJNA336433	SAMN05510606	2015	Australia	Unknown	vanA	A1	Human
E0156	PRJNA336433	SAMN05510607	2015	Australia	Unknown	vanB	A1	Human
E0157	PRJNA336433	SAMN05510608	2015	Australia	Unknown	vanB	A1	Human
E0158	PRJNA336433	SAMN05510609	2015	Australia	Unknown	vanB	A1	Human

E0159	PRJNA336433	SAMN05510610	2015	Australia	Unknown	vanB	A1	Human
E0160	PRJNA336433	SAMN05510611	2015	Australia	Unknown	vanB	A1	Human
E0161	PRJNA336433	SAMN05510612	2015	Australia	Unknown	vanB	A1	Human
E0162	PRJNA336433	SAMN05510613	2015	Australia	Unknown	vanB	A1	Human
E0163	PRJNA336433	SAMN05510614	2015	Australia	Unknown	vanB	A1	Human
E0164	PRJNA336433	SAMN05510615	2015	Australia	Unknown	vanB	A1	Human
E0165	PRJNA336433	SAMN05510616	2015	Australia	Unknown	vanB	A1	Human
E0166	PRJNA336433	SAMN05510617	2015	Australia	Unknown	vanB	A1	Human
E0167	PRJNA336433	SAMN05510618	2015	Australia	Unknown	vanB	A1	Human
E0168	PRJNA336433	SAMN05510619	2015	Australia	Unknown	vanB	A1	Human
E0169	PRJNA336433	SAMN05510620	2015	Australia	Unknown	vanB	A1	Human
E0170	PRJNA336433	SAMN05510621	2015	Australia	Unknown	vanB	A1	Human
E0171	PRJNA336433	SAMN05510622	2015	Australia	Unknown	vanB	A1	Human
E0172	PRJNA336433	SAMN05510623	2015	Australia	Unknown	vanB	A1	Human
E0173	PRJNA336433	SAMN05510624	2015	Australia	Unknown	vanA	A1	Human
E0174	PRJNA336433	SAMN05510625	2015	Australia	Unknown	vanB	A1	Human
E0175	PRJNA336433	SAMN05510626	2015	Australia	Unknown	vanB	A1	Human
E0176	PRJNA336433	SAMN05510627	2015	Australia	Unknown	vanB	A1	Human
E0177	PRJNA336433	SAMN05510628	2015	Australia	Unknown	vanA	A1	Human
E0178	PRJNA336433	SAMN05510629	2015	Australia	Unknown	vanB	A1	Human
E0179	PRJNA336433	SAMN05510630	2015	Australia	Unknown	vanB	A1	Human
E0180	PRJNA336433	SAMN05510631	2015	Australia	Unknown	vanB	A1	Human
E0181	PRJNA336433	SAMN05510632	2015	Australia	Unknown	vanB	A1	Human
E0182	PRJNA336433	SAMN05510633	2015	Australia	Unknown	vanB	A1	Human
E0183	PRJNA336433	SAMN05510634	2015	Australia	Unknown	vanB	A1	Human
E0184	PRJNA336433	SAMN05510635	2015	Australia	Unknown	vanB	A1	Human
E0185	PRJNA336433	SAMN05510636	2015	Australia	Unknown	vanA	A1	Human

E0186	PRJNA336433	SAMN05510637	2015	Australia	Unknown	vanB	A1	Human
E0187	PRJNA336433	SAMN05510638	2015	Australia	Unknown	vanB	A1	Human
E0188	PRJNA336433	SAMN05510639	2015	Australia	Unknown	vanB	A1	Human
E0189	PRJNA336433	SAMN05510640	2015	Australia	Unknown	vanB	A1	Human
E0190	PRJNA336433	SAMN05510641	2015	Australia	Unknown	vanB	A1	Human
E0191	PRJNA336433	SAMN05510642	2015	Australia	Unknown	vanB	A1	Human
E0192	PRJNA336433	SAMN05510643	2015	Australia	Unknown	vanB	A1	Human
E0193	PRJNA336433	SAMN05510644	2015	Australia	Unknown	vanB	A1	Human
E0194	PRJNA336433	SAMN05510645	2015	Australia	Unknown	vanB	A1	Human
E0195	PRJNA336433	SAMN05510646	2015	Australia	Unknown	vanB	A1	Human
E0196	PRJNA205886	SAMN02318071	1999	Australia	Infection	vanB	A1	Human
E0197	PRJNA205886	SAMN02318157	1999	Australia	Infection	None	A1	Human
E0198	PRJNA205886	SAMN02318072	2000	Australia	Infection	None	A2	Human
E0199	PRJNA205886	SAMN02318158	2000	Australia	Infection	vanB	A1	Human
E0200	PRJNA205886	SAMN02318073	2002	Australia	Infection	None	A1	Human
E0201	PRJNA205886	SAMN02318074	2003	Australia	Infection	vanB	A1	Human
E0202	PRJNA205886	SAMN02318075	2003	Australia	Infection	None	A1	Human
E0203	PRJNA205886	SAMN02318159	2004	Australia	Infection	None	A1	Human
E0204	PRJNA205886	SAMN02318076	2004	Australia	Infection	vanB	A1	Human
E0205	PRJNA205886	SAMN02318077	2005	Australia	Infection	None	A1	Human
E0206	PRJNA205886	SAMN02318078	2005	Australia	Infection	vanB	A1	Human
E0207	PRJNA205886	SAMN02318160	2005	Australia	Infection	vanB	A1	Human
E0208	PRJNA205886	SAMN02318079	2006	Australia	Infection	None	A1	Human
E0209	PRJNA205886	SAMN02318161	2006	Australia	Infection	None	A1	Human
E0210	PRJNA205886	SAMN02318080	2006	Australia	Infection	None	A1	Human
E0211	PRJNA205886	SAMN02318162	2006	Australia	Infection	None	A1	Human
E0212	PRJNA205886	SAMN02318163	2007	Australia	Infection	vanB	A1	Human

E0213	PRJNA205886	SAMN02318081	2007	Australia	Infection	vanB	A1	Human
E0214	PRJNA205886	SAMN02318164	2007	Australia	Infection	vanB	A1	Human
E0215	PRJNA205886	SAMN02318166	2007	Australia	Infection	None	A1	Human
E0216	PRJNA205886	SAMN02318167	2007	Australia	Infection	vanB	A1	Human
E0217	PRJNA205886	SAMN02318169	2007	Australia	Infection	None	A1	Human
E0218	PRJNA205886	SAMN02318170	2008	Australia	Infection	None	A1	Human
E0219	PRJNA205886	SAMN02318171	2008	Australia	Infection	None	A1	Human
E0220	PRJNA205886	SAMN02318172	2008	Australia	Infection	None	A1	Human
E0221	PRJNA205886	SAMN02318174	2008	Australia	Infection	vanB	A1	Human
E0222	PRJNA205886	SAMN02318175	2008	Australia	Infection	vanB	A1	Human
E0223	PRJNA205886	SAMN02318176	2008	Australia	Infection	vanB	A1	Human
E0224	PRJNA205886	SAMN02318180	2008	Australia	Infection	None	A1	Human
E0225	PRJNA205886	SAMN02318181	2008	Australia	Infection	None	A1	Human
E0226	PRJNA205886	SAMN02318182	2009	Australia	Infection	vanB	A1	Human
E0227	PRJNA205886	SAMN02318183	2009	Australia	Infection	vanB	A1	Human
E0228	PRJNA205886	SAMN02318185	2009	Australia	Infection	vanB	A1	Human
E0229	PRJNA205886	SAMN02318082	2009	Australia	Infection	vanB	A1	Human
E0230	PRJNA205886	SAMN02318188	1999	Australia	Infection	None	A2	Human
E0231	PRJNA205886	SAMN02318084	2009	Australia	Infection	None	A1	Human
E0232	PRJNA205886	SAMN02318087	2003	Australia	Infection	None	A1	Human
E0233	PRJNA205886	SAMN02318088	2000	Australia	Infection	None	A1	Human
E0234	PRJNA205886	SAMN02318089	2000	Australia	Infection	None	A2	Human
E0235	PRJNA205886	SAMN02318091	2010	Australia	Screening	None	A1	Human
E0236	PRJNA205886	SAMN02318189	2010	Australia	Screening	vanB	A1	Human
E0237	PRJNA205886	SAMN02318092	2010	Australia	Screening	vanB	A1	Human
E0238	PRJEB28731	SAMEA5367834	2015	Denmark	Screening	vanA	A1	Human
E0239	PRJEB28731	SAMEA5367835	2015	Denmark	Screening	vanA	A1	Human

E0240	PRJEB28731	SAMEA5367854	2015	Denmark	Screening	vanA	A1	Human
E0241	PRJEB28731	SAMEA5367873	2015	Denmark	Screening	vanA	A1	Human
E0242	PRJEB28731	SAMEA5367874	2015	Denmark	Screening	vanA	A1	Human
E0243	PRJEB28731	SAMEA5367880	2015	Denmark	Screening	vanA	A1	Human
E0244	PRJEB28731	SAMEA5367909	2015	Denmark	Screening	vanA	A1	Human
E0245	PRJEB28731	SAMEA5367918	2015	Denmark	Screening	vanA	A1	Human
E0246	PRJEB28731	SAMEA5367922	2013	Denmark	Screening	vanA	A1	Human
E0247	PRJEB28731	SAMEA4937746	2015	Denmark	Screening	vanA	A1	Human
E0248	PRJEB28731	SAMEA5367953	2015	Denmark	Screening	vanA	A1	Human
E0249	PRJNA636894	SAMN15088128	2015	Denmark	Infection	vanA	A1	Human
E0250	PRJEB28731	SAMEA5367998	2015	Denmark	Infection	vanA	A1	Human
E0251	PRJEB28731	SAMEA5368005	2013	Denmark	Screening	vanA	A1	Human
E0252	PRJEB28731	SAMEA5368007	2013	Denmark	Screening	vanA	A1	Human
E0253	PRJEB28731	SAMEA5368009	2013	Denmark	Screening	vanA	A1	Human
E0254	PRJEB28731	SAMEA5368026	2013	Denmark	Infection	vanA	A1	Human
E0255	PRJEB28731	SAMEA5368039	2013	Denmark	Infection	vanA	A1	Human
E0256	PRJEB28731	SAMEA5368041	2013	Denmark	Screening	vanA	A1	Human
E0257	PRJEB28731	SAMEA5368053	2013	Denmark	Infection	vanA	A1	Human
E0258	PRJEB14640	SAMEA4052470	2012	Denmark	Infection	vanA	A2	Human
E0259	PRJEB28731	SAMEA5368059	2013	Denmark	Infection	vanA	A1	Human
E0260	PRJEB28731	SAMEA5368060	2013	Denmark	Infection	vanA	A1	Human
E0261	PRJEB28731	SAMEA5368061	2013	Denmark	Infection	vanA	A1	Human
E0262	PRJEB28731	SAMEA4937740	2012	Denmark	Infection	vanA	A1	Human
E0263	PRJEB14636	SAMEA4052466	2013	Denmark	Screening	vanA	A1	Human
E0264	PRJEB28731	SAMEA5368098	2013	Denmark	Screening	vanA	A1	Human
E0265	PRJEB28731	SAMEA5368114	2014	Denmark	Infection	vanA	A1	Human
E0266	PRJEB28731	SAMEA5368119	2014	Denmark	Screening	vanA	A1	Human

E0267	PRJEB14632	SAMEA4052462	2014	Denmark	Screening	vanA	A1	Human
E0268	PRJEB28731	SAMEA5368154	2014	Denmark	Screening	vanA	A1	Human
E0269	PRJEB28731	SAMEA5368158	2012	Denmark	Infection	vanA	A1	Human
E0270	PRJEB28731	SAMEA5368159	2014	Denmark	Infection	vanA	A1	Human
E0271	PRJNA636894	SAMN15088129	2014	Denmark	Screening	vanA	A1	Human
E0272	PRJEB14635	SAMEA4052465	2014	Denmark	Screening	vanA	A1	Human
E0273	PRJEB28731	SAMEA5368193	2013	Denmark	Screening	vanA	A1	Human
E0274	PRJEB28731	SAMEA5368210	2014	Denmark	Infection	vanA	A1	Human
E0275	PRJEB14634	SAMEA4052464	2014	Denmark	Screening	vanA	A1	Human
E0276	PRJEB28731	SAMEA5368212	2014	Denmark	Screening	vanA	A1	Human
E0277	PRJEB28731	SAMEA5368221	2014	Denmark	Infection	vanA	A1	Human
E0278	PRJEB28731	SAMEA5368225	2014	Denmark	Screening	vanA	A1	Human
E0279	PRJEB28731	SAMEA4937742	2014	Denmark	Screening	vanA	A2	Human
E0280	PRJEB28731	SAMEA5368253	2014	Denmark	Screening	vanA	A1	Human
E0281	PRJEB14639	SAMEA4052469	2013	Denmark	Infection	vanA	A1	Human
E0282	PRJEB28731	SAMEA5368269	2014	Denmark	Screening	vanA	A1	Human
E0283	PRJEB14631	SAMEA4052461	2014	Denmark	Screening	vanA	A1	Human
E0284	PRJEB28731	SAMEA5368289	2014	Denmark	Screening	vanA	A1	Human
E0285	PRJEB28731	SAMEA5368296	2014	Denmark	Infection	vanA	A1	Human
E0286	PRJEB28731	SAMEA5368310	2014	Denmark	Screening	vanA	A1	Human
E0287	PRJEB28731	SAMEA5368318	2014	Denmark	Infection	vanA	A1	Human
E0288	PRJEB14629	SAMEA4052459	2014	Denmark	Infection	vanA	A1	Human
E0289	PRJEB28731	SAMEA5368352	2015	Denmark	Screening	vanA	A1	Human
E0290	PRJEB28731	SAMEA5368355	2013	Denmark	Screening	vanA	A1	Human
E0291	PRJEB28731	SAMEA5368386	2013	Denmark	Infection	vanA	A1	Human
E0292	PRJEB28731	SAMEA5368388	2014	Denmark	Infection	vanA	A1	Human
E0293	PRJEB28731	SAMEA5368420	2015	Denmark	Screening	vanA	A1	Human

E0294	PRJEB28731	SAMEA5368487	2014	Denmark	Infection	vanA	A1	Human
E0295	PRJEB28731	SAMEA5368529	2015	Denmark	Screening	vanA	A1	Human
E0296	PRJEB28731	SAMEA5368543	2015	Denmark	Screening	vanA	A1	Human
E0297	PRJEB28731	SAMEA5368570	2015	Denmark	Screening	vanA	A1	Human
E0298	PRJEB14633	SAMEA4052463	2013	Denmark	Screening	vanA	A1	Human
E0299	PRJEB28731	SAMEA5368631	2015	Denmark	Screening	vanA	A1	Human
E0300	PRJNA636894	SAMN15088130	2013	Denmark	Infection	None	B	Human
E0301	PRJEB28731	SAMEA5368636	2013	Denmark	Infection	None	B	Human
E0302	PRJEB28731	SAMEA5368654	2013	Denmark	Infection	None	A1	Human
E0303	PRJNA636894	SAMN15088131	2013	Denmark	Infection	None	A1	Human
E0304	PRJEB28731	SAMEA5368657	2013	Denmark	Infection	None	A1	Human
E0305	PRJEB28731	SAMEA5368664	2013	Denmark	Infection	None	A1	Human
E0306	PRJEB28731	SAMEA5368668	2013	Denmark	Infection	None	A2	Human
E0307	PRJEB28731	SAMEA5368670	2013	Denmark	Infection	None	A1	Human
E0308	PRJNA636894	SAMN15088132	2014	Denmark	Infection	None	A2	Human
E0309	PRJEB28731	SAMEA5368685	2013	Denmark	Infection	None	A1	Human
E0310	PRJNA636894	SAMN15088133	2013	Denmark	Infection	None	A2	Human
E0311	PRJNA636894	SAMN15088134	2014	Denmark	Infection	None	A2	Human
E0312	PRJNA636894	SAMN15088135	2013	Denmark	Infection	None	A1	Human
E0313	PRJNA636894	SAMN15088136	2013	Denmark	Infection	None	A2	Human
E0314	PRJEB28731	SAMEA5368708	2015	Denmark	Infection	None	A1	Human
E0315	PRJEB28731	SAMEA5368710	2015	Denmark	Infection	None	B	Human
E0316	PRJEB28731	SAMEA5368713	2015	Denmark	Infection	None	A1	Human
E0317	PRJEB28731	SAMEA5368717	2015	Denmark	Infection	None	A1	Human
E0318	PRJEB28731	SAMEA5368718	2015	Denmark	Infection	None	A2	Human
E0319	PRJEB28731	SAMEA5368735	2015	Denmark	Infection	None	A1	Human
E0320	PRJEB28731	SAMEA5368738	2015	Denmark	Infection	None	A1	Human

E0321	PRJEB28731	SAMEA5368742	2014	Denmark	Infection	None	A1	Human
E0322	PRJNA636894	SAMN15088137	2013	Denmark	Infection	None	B	Human
E0323	PRJNA636894	SAMN15088138	2013	Denmark	Infection	None	B	Human
E0324	PRJNA636894	SAMN15088139	2013	Denmark	Infection	None	A2	Human
E0325	PRJNA636894	SAMN15088140	2013	Denmark	Infection	None	A2	Human
E0326	PRJEB28731	SAMEA5368760	2013	Denmark	Infection	None	A2	Human
E0327	PRJNA636894	SAMN15088141	2013	Denmark	Infection	None	A1	Human
E0328	PRJNA636894	SAMN15088142	2013	Denmark	Infection	None	A2	Human
E0329	PRJNA636894	SAMN15088143	2015	Denmark	Infection	vanA	A1	Human
E0330	PRJNA636894	SAMN15088144	2015	Denmark	Infection	vanA	A1	Human
E0331	PRJEB38219	SAMEA6819264	2015	Denmark	Infection	vanA	A1	Human
E0332	PRJNA636894	SAMN15088145	2015	Denmark	Infection	vanA	A1	Human
E0333	PRJNA636894	SAMN15088146	2015	Denmark	Infection	vanA	A1	Human
E0334	PRJEB38219	SAMEA6819544	2015	Denmark	Infection	vanA	A1	Human
E0335	PRJEB38219	SAMEA6819454	2015	Denmark	Infection	vanA	A1	Human
E0336	PRJEB38219	SAMEA6819337	2015	Denmark	Infection	vanA	A1	Human
E0337	PRJNA636894	SAMN15088147	2015	Denmark	Infection	vanA	A1	Human
E0338	PRJEB38219	SAMEA6819260	2015	Denmark	Infection	vanA	A1	Human
E0339	PRJEB38219	SAMEA6819340	2015	Denmark	Infection	vanA	A1	Human
E0340	PRJEB38219	SAMEA6819398	2015	Denmark	Infection	vanA	A1	Human
E0341	PRJNA636894	SAMN15088148	2015	Denmark	Infection	vanA	A1	Human
E0342	PRJNA636894	SAMN15088149	2015	Denmark	Infection	vanB	A1	Human
E0343	PRJEB38219	SAMEA6819435	2015	Denmark	Infection	vanA	A2	Human
E0344	PRJEB38219	SAMEA6819356	2015	Denmark	Infection	vanA	A1	Human
E0345	PRJNA636894	SAMN15088150	2015	Denmark	Infection	vanA	A1	Human
E0346	PRJEB38219	SAMEA6819408	2015	Denmark	Infection	vanA	A1	Human
E0347	PRJNA636894	SAMN15088151	2015	Denmark	Infection	vanA	A1	Human

E0348	PRJEB38219	SAMEA6819348	2015	Denmark	Infection	vanA	A1	Human
E0349	PRJEB38219	SAMEA6819527	2015	Denmark	Infection	vanA	A1	Human
E0350	PRJEB38219	SAMEA6819333	2015	Denmark	Infection	vanA	A1	Human
E0351	PRJEB38219	SAMEA6819461	2015	Denmark	Infection	vanA	A1	Human
E0352	PRJNA636894	SAMN15088152	2015	Denmark	Infection	vanA	A1	Human
E0353	PRJEB38219	SAMEA6819416	2015	Denmark	Infection	vanA	A1	Human
E0354	PRJNA636894	SAMN15088153	2015	Denmark	Infection	vanA	A1	Human
E0355	PRJEB38219	SAMEA6819534	2015	Denmark	Infection	vanA	A1	Human
E0356	PRJNA636894	SAMN15088154	2015	Denmark	Infection	vanA	A1	Human
E0357	PRJEB38219	SAMEA6819368	2015	Denmark	Infection	vanA	A1	Human
E0358	PRJNA636894	SAMN15088155	2015	Denmark	Infection	vanA	A1	Human
E0359	PRJEB38219	SAMEA6819411	2015	Denmark	Infection	vanA	A1	Human
E0360	PRJEB38219	SAMEA6819563	2015	Denmark	Infection	vanA	A1	Human
E0361	PRJEB38219	SAMEA6819509	2015	Denmark	Infection	vanA	A1	Human
E0362	PRJEB38219	SAMEA6819501	2015	Denmark	Infection	vanA	A1	Human
E0363	PRJNA636894	SAMN15088156	2012	Norway	Infection	None	A1	Human
E0364	PRJNA39325	SAMN07326774	2012	India	Screening	None	A2	Human
E0365	PRJNA636894	SAMN15088157	2014	Norway	Infection	None	A1	Human
E0366	PRJNA636894	SAMN15088158	2012	Norway	Infection	None	A1	Human
E0367	PRJNA636894	SAMN15088159	2015	Norway	Infection	None	A1	Human
E0368	PRJNA636894	SAMN15088160	2014	Norway	Infection	None	A1	Human
E0369	PRJNA636894	SAMN15088161	2015	India	Screening	None	A2	Human
E0370	PRJNA486083	SAMN09836111	2015	Norway	Infection	None	A1	Human
E0371	PRJNA486083	SAMN09836113	2015	Norway	Infection	None	A1	Human
E0372	PRJNA636894	SAMN15088162	2015	Norway	Infection	None	A1	Human
E0373	PRJNA636894	SAMN15088163	2015	Norway	Infection	None	A1	Human
E0374	PRJNA636894	SAMN15088164	2015	Norway	Infection	None	A1	Human

E0375	PRJNA636894	SAMN15088165	2015	Norway	Infection	None	A1	Human
E0376	PRJNA636894	SAMN15088166	2015	Norway	Screening	None	A1	Human
E0377	PRJNA306646	SAMN04358604	2013	Norway	Infection	None	A1	Human
E0378	PRJNA306646	SAMN04358603	2013	Norway	Infection	None	A1	Human
E0379	PRJNA306646	SAMN04358606	2014	Norway	Infection	None	A1	Human
E0380	PRJNA306646	SAMN04358605	2014	Norway	Infection	None	A1	Human
E0381	PRJNA306646	SAMN04358607	2014	Norway	Screening	None	A1	Human
E0382	PRJNA306646	SAMN04358608	2014	Norway	Screening	None	A1	Human
E0383	PRJNA551094	SAMN12138986	2014	Sweden	Screening	None	A1	Human
E0384	PRJEB4345	SAMEA2156170	2012	UK	Infection	None	A1	Human
E0385	PRJEB4345	SAMEA2151512	2007	UK	Infection	None	A1	Human
E0386	PRJEB4345	SAMEA2160738	2010	UK	Infection	None	A1	Human
E0387	PRJEB4345	SAMEA2155053	2007	UK	Infection	vanA	A1	Human
E0388	PRJEB4345	SAMEA2144371	2007	UK	Infection	vanA	A1	Human
E0389	PRJEB4345	SAMEA2144485	2007	UK	Infection	None	A1	Human
E0390	PRJEB4345	SAMEA2144849	2009	UK	Infection	vanA	A1	Human
E0391	PRJEB4345	SAMEA2148075	2009	UK	Infection	None	A1	Human
E0392	PRJEB4345	SAMEA2152857	2007	UK	Infection	vanA	A1	Human
E0393	PRJEB4345	SAMEA2153652	2010	UK	Infection	vanA	A1	Human
E0394	PRJEB4345	SAMEA2160445	2007	UK	Infection	vanA	A1	Human
E0395	PRJEB4345	SAMEA2145396	2012	UK	Infection	vanA	A1	Human
E0396	PRJEB4345	SAMEA2150716	2008	UK	Infection	vanA	A1	Human
E0397	PRJEB4345	SAMEA2161968	2009	UK	Infection	None	A1	Human
E0398	PRJEB4345	SAMEA2149661	2010	UK	Infection	vanA	A1	Human
E0399	PRJEB4345	SAMEA2154328	2007	UK	Infection	vanA	A1	Human
E0400	PRJEB4345	SAMEA2147944	2012	UK	Infection	None	A1	Human
E0401	PRJEB4345	SAMEA2162849	2006	UK	Infection	vanA	A1	Human

E0402	PRJEB4345	SAMEA2146127	2010	UK	Infection	None	A1	Human
E0403	PRJEB4345	SAMEA2158947	2012	UK	Infection	None	A1	Human
E0404	PRJEB4345	SAMEA2152475	2008	UK	Infection	None	A1	Human
E0405	PRJEB4345	SAMEA2155160	2008	UK	Infection	vanA	A1	Human
E0406	PRJEB4345	SAMEA2154026	2008	UK	Infection	None	A1	Human
E0407	PRJEB4345	SAMEA2148195	2012	UK	Infection	None	A1	Human
E0408	PRJEB4345	SAMEA2150825	2008	UK	Infection	None	A1	Human
E0409	PRJEB4345	SAMEA2158563	2010	UK	Infection	None	A1	Human
E0410	PRJEB4345	SAMEA2146589	2008	UK	Infection	vanA	A1	Human
E0411	PRJEB4345	SAMEA2147386	2009	UK	Infection	vanA	A1	Human
E0412	PRJEB4345	SAMEA2143510	2009	UK	Infection	vanA	A1	Human
E0413	PRJEB4345	SAMEA2145560	2006	UK	Infection	None	A1	Human
E0414	PRJEB4345	SAMEA1967440	2009	UK	Infection	None	A1	Human
E0415	PRJEB4345	SAMEA2148477	2010	UK	Infection	None	A1	Human
E0416	PRJEB4345	SAMEA2155069	2012	UK	Infection	vanA	A1	Human
E0417	PRJEB4345	SAMEA2160746	2009	UK	Infection	vanA	A1	Human
E0418	PRJEB4345	SAMEA2149301	2010	UK	Infection	vanA	A1	Human
E0419	PRJEB4345	SAMEA2160311	2012	UK	Infection	vanB	A1	Human
E0420	PRJEB4345	SAMEA2155593	2009	UK	Infection	vanA	A1	Human
E0421	PRJEB4345	SAMEA2145781	2009	UK	Infection	None	A1	Human
E0422	PRJEB4345	SAMEA2162288	2007	UK	Infection	vanA	A1	Human
E0423	PRJEB4345	SAMEA2148590	2012	UK	Infection	vanA	A1	Human
E0424	PRJEB4345	SAMEA1969798	2010	UK	Infection	None	A1	Human
E0425	PRJEB4345	SAMEA2145390	2011	UK	Infection	None	A1	Human
E0426	PRJEB4345	SAMEA2163506	2009	UK	Infection	vanA	A1	Human
E0427	PRJEB4345	SAMEA2147347	2007	UK	Infection	vanA	A1	Human
E0428	PRJEB4345	SAMEA2162516	2011	UK	Infection	vanA	A1	Human

E0429	PRJEB4345	SAMEA2163343	2011	UK	Infection	None	A1	Human
E0430	PRJEB4345	SAMEA2148769	2010	UK	Infection	None	A1	Human
E0431	PRJEB4345	SAMEA2151254	2006	UK	Infection	vanA	A1	Human
E0432	PRJEB4345	SAMEA2156302	2011	UK	Infection	vanA	A1	Human
E0433	PRJEB4345	SAMEA2158364	2010	UK	Infection	vanA	A1	Human
E0434	PRJEB4345	SAMEA2148623	2006	UK	Infection	vanA	A1	Human
E0435	PRJEB4345	SAMEA2155441	2010	UK	Infection	vanA	A1	Human
E0436	PRJEB4345	SAMEA2148636	2009	UK	Infection	vanA	A1	Human
E0437	PRJEB4345	SAMEA2145117	2009	UK	Infection	None	A1	Human
E0438	PRJEB4345	SAMEA2153486	2008	UK	Infection	vanA	A1	Human
E0439	PRJEB4345	SAMEA2146844	2010	UK	Infection	None	A1	Human
E0440	PRJEB4345	SAMEA2142474	2009	UK	Infection	vanA	A1	Human
E0441	PRJEB4345	SAMEA1972111	2007	UK	Infection	vanA	A1	Human
E0442	PRJEB4345	SAMEA2154532	2012	UK	Infection	vanA	A1	Human
E0443	PRJEB4345	SAMEA2153388	2008	UK	Infection	vanA	A1	Human
E0444	PRJEB4345	SAMEA2153215	2008	UK	Infection	None	A1	Human
E0445	PRJEB4345	SAMEA2143765	2006	UK	Infection	None	A1	Human
E0446	PRJEB4345	SAMEA2157371	2009	UK	Infection	vanA	A1	Human
E0447	PRJEB4345	SAMEA2154769	2007	UK	Infection	vanB	A1	Human
E0448	PRJEB4345	SAMEA2154569	2011	UK	Infection	vanA	A1	Human
E0449	PRJEB4345	SAMEA2158662	2007	UK	Infection	None	A1	Human
E0450	PRJEB4345	SAMEA2149996	2008	UK	Infection	None	A1	Human
E0451	PRJEB4345	SAMEA2157045	2009	UK	Infection	None	A1	Human
E0452	PRJEB4345	SAMEA2159966	2012	UK	Infection	vanA	A1	Human
E0453	PRJEB4345	SAMEA2143077	2011	UK	Infection	None	A1	Human
E0454	PRJEB4345	SAMEA2154297	2011	UK	Infection	None	A1	Human
E0455	PRJEB4345	SAMEA2155476	2008	UK	Infection	None	A1	Human

E0456	PRJEB4345	SAMEA2149130	2007	UK	Infection	None	A1	Human
E0457	PRJEB4345	SAMEA2147574	2009	UK	Infection	vanA	A1	Human
E0458	PRJEB4345	SAMEA2150484	2012	UK	Infection	vanB	A1	Human
E0459	PRJEB4345	SAMEA2151753	2012	UK	Infection	None	A1	Human
E0460	PRJEB4345	SAMEA2144936	2012	UK	Infection	None	A1	Human
E0461	PRJEB4345	SAMEA2152523	2011	UK	Infection	vanA	A1	Human
E0462	PRJEB4345	SAMEA2152856	2011	UK	Infection	None	A1	Human
E0463	PRJEB4345	SAMEA2148286	2012	UK	Infection	None	A1	Human
E0464	PRJEB4345	SAMEA2155647	2010	UK	Infection	vanA	A1	Human
E0465	PRJEB4345	SAMEA2146613	2011	UK	Infection	vanA	A1	Human
E0466	PRJEB4345	SAMEA2154647	2008	UK	Infection	vanA	A1	Human
E0467	PRJEB4345	SAMEA2145669	2012	UK	Infection	None	A1	Human
E0468	PRJEB4345	SAMEA2152547	2007	UK	Infection	vanA	A1	Human
E0469	PRJEB4345	SAMEA2147837	2007	UK	Infection	vanA	A1	Human
E0470	PRJEB4345	SAMEA2156326	2008	UK	Infection	None	A1	Human
E0471	PRJEB4345	SAMEA2163546	2012	UK	Infection	None	A1	Human
E0472	PRJEB4345	SAMEA2146884	2010	UK	Infection	vanA	A1	Human
E0473	PRJEB4345	SAMEA2159244	2011	UK	Infection	None	A1	Human
E0474	PRJEB4345	SAMEA2149979	2010	UK	Infection	None	A1	Human
E0475	PRJEB4345	SAMEA2146967	2011	UK	Infection	vanA	A1	Human
E0476	PRJEB4345	SAMEA2154441	2012	UK	Infection	None	A1	Human
E0477	PRJEB4345	SAMEA1969240	2007	UK	Infection	None	A1	Human
E0478	PRJEB4345	SAMEA2142832	2009	UK	Infection	vanA	A1	Human
E0479	PRJEB4345	SAMEA2146840	2008	UK	Infection	vanA	A1	Human
E0480	PRJEB4345	SAMEA2151772	2009	UK	Infection	vanA	A1	Human
E0481	PRJEB4345	SAMEA2150875	2011	UK	Infection	vanA	A1	Human
E0482	PRJEB4345	SAMEA2161933	2010	UK	Infection	vanA	A1	Human

E0483	PRJEB4345	SAMEA2157054	2011	UK	Infection	vanA	A1	Human
E0484	PRJNA88329	SAMN00808981	1999	Netherlands	Screening	None	A2	Human
E0485	PRJNA88321	SAMN00808976	1995	Netherlands	Screening	None	A1	Human
E0486	PRJNA88349	SAMN00808992	1994	USA	Screening	None	A2	Human
E0487	PRJNA88805	SAMN00809130	1996	USA	Screening	None	A1	Human
E0488	PRJNA89009	SAMN00809200	1996	USA	Screening	None	A1	Human
E0489	PRJNA88345	SAMN00809201	1993	USA	Unknown	None	A1	Human
E0490	PRJNA88349	SAMN00808989	1994	USA	Unknown	None	A1	Human
E0491	PRJNA88355	SAMN00808995	1996	France	Unknown	None	A1	Human
E0492	PRJNA88357	SAMN00808996	1996	Switzerland	Unknown	None	A1	Human
E0493	PRJNA88381	SAMN00809009	1996	Switzerland	Unknown	None	A1	Human
E0494	PRJNA88385	SAMN00809011	2010	Denmark	Screening	None	A1	Human
E0495	PRJNA88379	SAMN00809008	1996	USA	Unknown	None	A1	Human
E0496	PRJNA88821	SAMN00809100	1996	France	Unknown	None	A1	Human
E0497	PRJNA88829	SAMN00809104	1992	Belgium	Infection	None	A1	Human
E0498	PRJNA73043	SAMN00779827	2013	Tanzania	Infection	None	A1	Human
E0499	PRJNA88375	SAMN00809006	2011	Denmark	Screening	None	A1	Human
E0500	PRJNA88801	SAMN00809108	1996	USA	Unknown	None	A1	Human
E0501	PRJNA88827	SAMN00809103	2000	France	Unknown	None	A1	Human
E0502	PRJNA88359	SAMN00808997	1996	USA	Unknown	None	A1	Human
E0503	PRJNA88475	SAMN00808918	1996	USA	Unknown	None	A1	Human
E0504	PRJNA88317	SAMN00808974	1995	Denmark	Screening	None	A1	Human
E0505	PRJNA88319	SAMN00808975	1995	Denmark	Screening	None	A1	Human
E0506	PRJNA88897	SAMN00809142	1986	France	Screening	None	A1	Human
E0507	PRJNA88437	SAMN00808917	1994	France	Unknown	None	A1	Human
E0508	PRJNA88351	SAMN00808993	1993	USA	Unknown	None	A1	Human
E0509	PRJNA88353	SAMN00808994	1993	USA	Unknown	None	A1	Human

E0510	PRJNA88817	SAMN00809098	1996	France	Unknown	None	A1	Human
E0511	PRJNA88819	SAMN00809099	1996	France	Unknown	None	A1	Human
E0512	PRJNA73043	SAMN00779806	2000	UK	Infection	None	A1	Human
E0513	PRJNA88383	SAMN00809010	1996	Switzerland	Unknown	None	A1	Human
E0514	PRJNA73043	SAMN00779846	1999	Italy	Infection	None	A1	Human
E0515	PRJNA73043	SAMN00779847	2002	Germany	Screening	None	A1	Human
E0516	PRJNA73043	SAMN00779848	2006	Netherlands	Infection	None	A1	Human
E0517	PRJNA73043	SAMN00779849	2002	Netherlands	Infection	None	A1	Human
E0518	PRJNA73043	SAMN00779851	2010	Latvia	Screening	None	A1	Human
E0519	PRJNA73043	SAMN00779852	2010	Portugal	Screening	None	A1	Human
E0520	PRJNA88325	SAMN00808978	1994	France	Unknown	None	A1	Human
E0521	PRJNA73043	SAMN00779844	1997	Israel	Infection	None	A1	Human
E0522	PRJNA88339	SAMN00808986	1994	France	Unknown	None	A1	Human
E0523	PRJNA88815	SAMN00809146	1996	Switzerland	Unknown	None	A1	Human
E0524	PRJNA73043	SAMN00779802	2001	USA	Screening	None	A1	Human
E0525	PRJNA73043	SAMN00779835	2001	USA	Infection	None	A1	Human
E0526	PRJNA88323	SAMN00808977	1994	France	Unknown	None	A1	Human
E0527	PRJNA88259	SAMN00808943	1995	Denmark	Screening	None	A1	Human
E0528	PRJNA88277	SAMN00808953	2003	Denmark	Screening	None	A1	Human
E0529	PRJNA88373	SAMN00809005	2010	Denmark	Infection	None	A1	Human
E0530	PRJNA88377	SAMN00809007	2010	Denmark	Infection	None	A1	Human
E0531	PRJNA89015	SAMN00809203	Unknown	USA	Screening	None	A1	Human
E0532	PRJNA89017	SAMN00809204	Unknown	USA	Infection	None	A1	Human
E0533	PRJNA89019	SAMN00809205	Unknown	USA	Screening	None	A1	Human
E0534	PRJNA89021	SAMN00809206	Unknown	USA	Infection	None	A1	Human
E0535	PRJNA89023	SAMN00809207	Unknown	USA	Screening	None	A1	Human
E0536	PRJNA89025	SAMN00809208	Unknown	USA	Infection	None	A1	Human

E0537	PRJNA89027	SAMN00809209	Unknown	USA	Screening	None	A1	Human
E0538	PRJNA73043	SAMN00779856	Unknown	USA	Screening	None	A1	Human
E0539	PRJNA73043	SAMN00779857	Unknown	USA	Infection	None	A1	Human
E0540	PRJNA73043	SAMN00779858	Unknown	USA	Infection	None	A1	Human
E0541	PRJNA193299	SAMN02604219	2009	Australia	Infection	None	A1	Human
E0542	PRJNA636894	SAMN15088167	1995	Netherlands	Infection	vanA	A2	Human
E0543	PRJEB28495	SAMEA4885994	1997	France	Screening	vanA	A2	Human
E0544	PRJNA636894	SAMN15088168	1997	Israel	Infection	vanA	A1	Human
E0545	PRJEB28495	SAMEA4884527	1997	France	Infection	None	A1	Human
E0546	PRJNA636894	SAMN15088169	Unknown	Italy	Infection	None	A1	Human
E0547	PRJEB28495	SAMEA4885997	Unknown	Poland	Infection	None	A2	Human
E0548	PRJEB28495	SAMEA4885998	Unknown	Austria	Infection	None	A1	Human
E0549	PRJNA636894	SAMN15088170	1998	France	Infection	None	A2	Human
E0550	PRJEB28495	SAMEA4886000	Unknown	Spain	Infection	None	A1	Human
E0551	PRJEB28495	SAMEA4886001	Unknown	Switzerland	Infection	None	A2	Human
E0552	PRJEB28495	SAMEA4886005	Unknown	Poland	Infection	None	A1	Human
E0553	PRJEB28495	SAMEA4884528	1999	Italy	Infection	None	A1	Human
E0554	PRJNA636894	SAMN15088171	2002	Netherlands	Screening	vanA	A2	Human
E0555	PRJNA636894	SAMN15088172	1957	Netherlands	Infection	None	A2	Human
E0556	PRJEB28495	SAMEA4886012	1998	Brazil	Screening	vanA	A2	Human
E0557	PRJEB28495	SAMEA4886015	2001	Tanzania	Infection	None	A2	Human
E0558	PRJNA636894	SAMN15088173	2001	Tanzania	Infection	None	A1	Human
E0559	PRJEB28495	SAMEA4886016	2000	Australia	Unknown	vanB	A1	Human
E0560	PRJNA636894	SAMN15088174	2001	Netherlands	Infection	None	A2	Human
E0561	PRJEB28495	SAMEA4886018	2004	Singapore	Infection	vanB	A1	Human
E0562	PRJNA636894	SAMN15088175	2005	Hungary	Infection	vanA	A1	Human
E0563	PRJNA636894	SAMN15088176	2002	Netherlands	Infection	None	A2	Human

E0564	PRJEB28495	SAMEA4884761	2010	Latvia	Screening	None	A1	Human
E0565	PRJEB28495	SAMEA4884765	2010	Latvia	Screening	None	A1	Human
E0566	PRJEB28495	SAMEA4884767	2010	Latvia	Infection	None	A1	Human
E0567	PRJEB28495	SAMEA4884805	2010	Portugal	Screening	None	A1	Human
E0568	PRJNA636894	SAMN15088177	2010	Italy	Screening	None	B	Human
E0569	PRJEB28495	SAMEA4884845	2010	Portugal	Screening	None	A1	Human
E0570	PRJEB28495	SAMEA4884854	2010	Latvia	Screening	None	A1	Human
E0571	PRJEB28495	SAMEA4884883	2010	Latvia	Screening	None	A2	Human
E0572	PRJEB28495	SAMEA4884887	2010	Latvia	Screening	None	A1	Human
E0573	PRJEB28495	SAMEA4884890	2009	France	Unknown	None	A1	Human
E0574	PRJEB28495	SAMEA4884893	2010	Slovenia	Unknown	None	A1	Human
E0575	PRJEB28495	SAMEA4884895	2009	France	Unknown	None	A1	Human
E0576	PRJEB28495	SAMEA4884931	2010	Portugal	Unknown	None	A1	Human
E0577	PRJEB28495	SAMEA4884936	2010	Italy	Unknown	None	A2	Human
E0578	PRJEB28495	SAMEA4884976	2008	Slovenia	Unknown	None	A1	Human
E0579	PRJEB28495	SAMEA4884977	2008	Slovenia	Unknown	None	A1	Human
E0580	PRJEB28495	SAMEA4885018	2009	Slovenia	Screening	None	A1	Human
E0581	PRJEB28495	SAMEA4885032	2010	Portugal	Screening	None	A1	Human
E0582	PRJEB28495	SAMEA4885042	2009	Luxembourg	Screening	None	A1	Human
E0583	PRJEB28495	SAMEA4885045	2009	Greece	Screening	None	A1	Human
E0584	PRJEB28495	SAMEA4885046	2009	Greece	Infection	None	A1	Human
E0585	PRJEB28495	SAMEA4885067	2010	Greece	Screening	None	A2	Human
E0586	PRJEB28495	SAMEA4885071	2010	Greece	Screening	None	A1	Human
E0587	PRJEB28495	SAMEA4885076	2010	Greece	Screening	None	A1	Human
E0588	PRJEB28495	SAMEA4885095	2009	Greece	Screening	None	A1	Human
E0589	PRJEB28495	SAMEA4885098	2008	Greece	Screening	None	A1	Human
E0590	PRJEB28495	SAMEA4885102	2009	Greece	Screening	vanA	A1	Human

E0591	PRJEB28495	SAMEA4885103	2009	Greece	Screening	None	A2	Human
E0592	PRJEB28495	SAMEA4885113	2012	Netherlands	Unknown	vanA	A1	Human
E0593	PRJEB28495	SAMEA4885117	2012	Netherlands	Unknown	vanA	A1	Human
E0594	PRJEB28495	SAMEA4885119	2012	Netherlands	Unknown	vanB	A1	Human
E0595	PRJEB28495	SAMEA4885181	Unknown	Netherlands	Unknown	vanA	A2	Human
E0596	PRJEB28495	SAMEA4885195	2012	Netherlands	Screening	vanA	A1	Human
E0597	PRJEB28495	SAMEA4885198	2012	Netherlands	Unknown	vanA	A1	Human
E0598	PRJEB28495	SAMEA4885207	2009	Netherlands	Infection	vanB	A1	Human
E0599	PRJEB28495	SAMEA4885211	2010	Netherlands	Infection	vanB	A1	Human
E0600	PRJEB28495	SAMEA4885217	2011	Netherlands	Screening	vanA	A1	Human
E0601	PRJEB28495	SAMEA4885232	2012	Netherlands	Unknown	vanA	A1	Human
E0602	PRJEB28495	SAMEA4885313	2012	Netherlands	Unknown	vanA	A2	Human
E0603	PRJEB28495	SAMEA4885317	2012	Netherlands	Unknown	vanA	A1	Human
E0604	PRJEB28495	SAMEA4885321	2012	Netherlands	Infection	vanB	A1	Human
E0605	PRJEB28495	SAMEA4885326	2012	Netherlands	Unknown	vanA	A1	Human
E0606	PRJEB28495	SAMEA4885331	2013	Netherlands	Infection	vanB	A1	Human
E0607	PRJEB28495	SAMEA4885333	2013	Netherlands	Screening	vanA	A2	Human
E0608	PRJEB28495	SAMEA4885339	2013	Netherlands	Screening	vanA	A1	Human
E0609	PRJEB28495	SAMEA4885369	2013	Netherlands	Screening	vanA	A1	Human
E0610	PRJEB28495	SAMEA4885382	2013	Netherlands	Screening	vanA	A2	Human
E0611	PRJEB28495	SAMEA4885407	2013	Netherlands	Unknown	vanB	A1	Human
E0612	PRJNA636894	SAMN15088178	2013	Netherlands	Unknown	vanA	B	Human
E0613	PRJEB28495	SAMEA4885413	2012	Netherlands	Unknown	vanB	A2	Human
E0614	PRJEB28495	SAMEA4885422	2013	Netherlands	Unknown	vanA	A1	Human
E0615	PRJEB28495	SAMEA4885462	2013	Netherlands	Unknown	vanB	A1	Human
E0616	PRJEB28495	SAMEA4885475	2013	Netherlands	Unknown	vanB	A1	Human
E0617	PRJEB28495	SAMEA4885490	2013	Netherlands	Screening	vanB	A1	Human

E0618	PRJEB28495	SAMEA4885498	2013	Netherlands	Unknown	vanA	A1	Human
E0619	PRJEB28495	SAMEA4885500	2013	Netherlands	Screening	vanA	A1	Human
E0620	PRJEB28495	SAMEA4885536	2014	Netherlands	Unknown	vanB	A1	Human
E0621	PRJEB28495	SAMEA4885543	2014	Netherlands	Unknown	vanB	A1	Human
E0622	PRJEB28495	SAMEA4885561	2014	Netherlands	Screening	vanA	A1	Human
E0623	PRJEB28495	SAMEA4885607	2014	Netherlands	Screening	vanA	A2	Human
E0624	PRJEB28495	SAMEA4885648	2005	Netherlands	Unknown	vanA	A1	Human
E0625	PRJEB28495	SAMEA4885669	2015	Netherlands	Unknown	vanB	A1	Human
E0626	PRJEB28495	SAMEA4885731	2015	Netherlands	Unknown	vanA	A1	Human
E0627	PRJEB28495	SAMEA4885734	2015	Netherlands	Unknown	vanA	A1	Human
E0628	PRJEB28495	SAMEA4885736	2015	Netherlands	Unknown	vanA	A1	Human
E0629	PRJEB28495	SAMEA4885768	2015	Netherlands	Unknown	vanA	A1	Human
E0630	PRJEB28495	SAMEA4885776	2015	Netherlands	Unknown	vanA	A1	Human
E0631	PRJEB28495	SAMEA4885781	2015	Netherlands	Unknown	vanA	A1	Human
E0632	PRJEB28495	SAMEA4885786	2015	Netherlands	Unknown	vanB	A1	Human
E0633	PRJEB28495	SAMEA4885796	2015	Netherlands	Unknown	vanB	A1	Human
E0634	PRJEB28495	SAMEA4885831	2015	Netherlands	Screening	vanC	A1	Human
E0635	PRJEB28495	SAMEA4885940	2015	Netherlands	Screening	vanA	A1	Human
E0636	PRJEB8624	SAMEA3388987	2003	Australia	Infection	None	A1	Human
E0637	PRJEB8624	SAMEA3388988	2012	Australia	Infection	None	A1	Human
E0638	PRJEB8624	SAMEA3388989	2004	Australia	Infection	None	A1	Human
E0639	PRJEB8624	SAMEA3388990	2012	Australia	Infection	vanB	A1	Human
E0640	PRJEB8624	SAMEA3388991	2006	Australia	Infection	None	A1	Human
E0641	PRJEB8624	SAMEA3388992	2012	Australia	Infection	None	A1	Human
E0642	PRJEB8624	SAMEA3388993	2007	Australia	Infection	vanB	A1	Human
E0643	PRJEB8624	SAMEA3388994	2013	Australia	Infection	None	A1	Human
E0644	PRJEB8624	SAMEA3388995	2011	Australia	Infection	vanB	A1	Human

E0645	PRJEB8624	SAMEA3388996	2012	Australia	Infection	None	A1	Human
E0646	PRJEB8624	SAMEA3388997	2011	Australia	Infection	vanB	A1	Human
E0647	PRJEB8624	SAMEA3388998	2012	Australia	Infection	None	A1	Human
E0648	PRJEB8624	SAMEA3388999	2011	Australia	Infection	None	A1	Human
E0649	PRJEB8624	SAMEA3389000	2013	Australia	Infection	None	A2	Human
E0650	PRJEB8624	SAMEA3389001	2012	Australia	Infection	None	A1	Human
E0651	PRJEB8624	SAMEA3389002	2012	Australia	Infection	vanB	A1	Human
E0652	PRJEB8624	SAMEA3389003	2004	Australia	Infection	None	A1	Human
E0653	PRJEB8624	SAMEA3389004	2012	Australia	Infection	None	A1	Human
E0654	PRJEB8624	SAMEA3389005	2012	Australia	Infection	vanB	A1	Human
E0655	PRJEB8624	SAMEA3389006	2006	Australia	Infection	None	A1	Human
E0656	PRJEB8624	SAMEA3389007	2012	Australia	Infection	vanB	A1	Human
E0657	PRJEB8624	SAMEA3389008	2007	Australia	Infection	None	A1	Human
E0658	PRJEB8624	SAMEA3389009	2013	Australia	Infection	None	A1	Human
E0659	PRJEB8624	SAMEA3389010	2011	Australia	Infection	None	A1	Human
E0660	PRJEB8624	SAMEA3389011	2012	Australia	Infection	None	A1	Human
E0661	PRJEB8624	SAMEA3389012	2011	Australia	Infection	None	A1	Human
E0662	PRJEB8624	SAMEA3389013	2013	Australia	Infection	None	A1	Human
E0663	PRJEB8624	SAMEA3389014	2011	Australia	Infection	vanB	A1	Human
E0664	PRJEB8624	SAMEA3389015	2012	Australia	Infection	None	A1	Human
E0665	PRJEB8624	SAMEA3389016	2012	Australia	Infection	vanB	A1	Human
E0666	PRJEB8624	SAMEA3389017	2012	Australia	Infection	None	A1	Human
E0667	PRJEB8624	SAMEA3389018	2005	Australia	Infection	None	A1	Human
E0668	PRJEB8624	SAMEA3389019	2012	Australia	Infection	None	A1	Human
E0669	PRJEB8624	SAMEA3389020	2006	Australia	Infection	None	A1	Human
E0670	PRJEB8624	SAMEA3389021	2012	Australia	Infection	None	A1	Human
E0671	PRJEB8624	SAMEA3389022	2013	Australia	Infection	None	A1	Human

E0672	PRJEB8624	SAMEA3389023	2011	Australia	Infection	None	A1	Human
E0673	PRJEB8624	SAMEA3389024	2013	Australia	Infection	None	A1	Human
E0674	PRJEB8624	SAMEA3389025	2011	Australia	Infection	vanB	A1	Human
E0675	PRJEB8624	SAMEA3389026	2012	Australia	Infection	None	A1	Human
E0676	PRJEB8624	SAMEA3389027	2011	Australia	Infection	None	A1	Human
E0677	PRJEB8624	SAMEA3389028	2012	Australia	Infection	vanB	A1	Human
E0678	PRJEB8624	SAMEA3389029	2012	Australia	Infection	vanB	A1	Human
E0679	PRJEB8624	SAMEA3389030	2004	Australia	Infection	None	A1	Human
E0680	PRJEB8624	SAMEA3389031	2012	Australia	Infection	None	A1	Human
E0681	PRJEB8624	SAMEA3389032	2007	Australia	Infection	None	A1	Human
E0682	PRJEB8624	SAMEA3389033	2012	Australia	Infection	None	A1	Human
E0683	PRJEB8624	SAMEA3389034	2006	Australia	Infection	vanB	A1	Human
E0684	PRJEB8624	SAMEA3389035	2012	Australia	Infection	None	A1	Human
E0685	PRJEB8624	SAMEA3389036	2007	Australia	Infection	None	A1	Human
E0686	PRJEB8624	SAMEA3389037	2013	Australia	Infection	None	A1	Human
E0687	PRJEB8624	SAMEA3389038	2011	Australia	Infection	vanB	A1	Human
E0688	PRJEB8624	SAMEA3389039	2013	Australia	Infection	None	A1	Human
E0689	PRJEB8624	SAMEA3389040	2011	Australia	Infection	vanB	A1	Human
E0690	PRJEB8624	SAMEA3389041	2012	Australia	Infection	vanB	A1	Human
E0691	PRJEB8624	SAMEA3389042	2011	Australia	Infection	None	A1	Human
E0692	PRJEB8624	SAMEA3389043	2012	Australia	Infection	vanB	A1	Human
E0693	PRJEB8624	SAMEA3389044	2012	Australia	Infection	vanB	A1	Human
E0694	PRJEB8624	SAMEA3389045	2012	Australia	Infection	vanB	A1	Human
E0695	PRJEB8624	SAMEA3389046	2004	Australia	Infection	vanB	A1	Human
E0696	PRJEB8624	SAMEA3389047	2011	Australia	Infection	None	A1	Human
E0697	PRJEB8624	SAMEA3389048	2005	Australia	Infection	vanB	A1	Human
E0698	PRJEB8624	SAMEA3389049	2006	Australia	Infection	None	A1	Human

E0699	PRJEB8624	SAMEA3389050	2012	Australia	Infection	vanB	A1	Human
E0700	PRJEB8624	SAMEA3389051	2007	Australia	Infection	None	A1	Human
E0701	PRJEB8624	SAMEA3389052	2013	Australia	Infection	None	A1	Human
E0702	PRJEB8624	SAMEA3389053	2011	Australia	Infection	None	A1	Human
E0703	PRJEB8624	SAMEA3389054	2013	Australia	Infection	vanB	A1	Human
E0704	PRJEB8624	SAMEA3389055	2011	Australia	Infection	None	A1	Human
E0705	PRJEB8624	SAMEA3389056	2012	Australia	Infection	None	A1	Human
E0706	PRJEB8624	SAMEA3389057	2011	Australia	Infection	vanB	A1	Human
E0707	PRJEB8624	SAMEA3389058	2012	Australia	Infection	None	A1	Human
E0708	PRJEB8624	SAMEA3389059	2012	Australia	Infection	vanB	A1	Human
E0709	PRJEB8624	SAMEA3389060	2012	Australia	Infection	None	A1	Human
E0710	PRJEB8624	SAMEA3389061	2004	Australia	Infection	None	A2	Human
E0711	PRJEB8624	SAMEA3389062	2013	Australia	Infection	None	A1	Human
E0712	PRJEB8624	SAMEA3389063	2005	Australia	Infection	vanB	A1	Human
E0713	PRJEB8624	SAMEA3389064	2012	Australia	Infection	None	A1	Human
E0714	PRJEB8624	SAMEA3389065	2007	Australia	Infection	vanB	A1	Human
E0715	PRJEB8624	SAMEA3389066	2012	Australia	Infection	vanB	A1	Human
E0716	PRJEB8624	SAMEA3389067	2005	Australia	Infection	vanB	A1	Human
E0717	PRJEB8624	SAMEA3389068	2013	Australia	Infection	None	A1	Human
E0718	PRJEB8624	SAMEA3389069	2011	Australia	Infection	None	A1	Human
E0719	PRJEB8624	SAMEA3389070	2011	Australia	Infection	None	A1	Human
E0720	PRJEB8624	SAMEA3389071	2011	Australia	Infection	vanB	A1	Human
E0721	PRJEB8624	SAMEA3389072	2012	Australia	Infection	None	A1	Human
E0722	PRJEB8624	SAMEA3389073	2004	Australia	Infection	None	A1	Human
E0723	PRJEB8624	SAMEA3389074	2005	Australia	Infection	vanB	A1	Human
E0724	PRJEB8624	SAMEA3389075	2007	Australia	Infection	vanB	A1	Human
E0725	PRJEB8624	SAMEA3389076	2007	Australia	Infection	vanB	A1	Human

E0726	PRJEB8624	SAMEA3389077	2011	Australia	Infection	None	A1	Human
E0727	PRJEB8624	SAMEA3389078	2011	Australia	Infection	vanB	A1	Human
E0728	PRJEB8624	SAMEA3389079	2011	Australia	Infection	None	A1	Human
E0729	PRJEB8624	SAMEA3389080	2012	Australia	Infection	None	A1	Human
E0730	PRJEB8624	SAMEA3389081	2004	Australia	Infection	None	A1	Human
E0731	PRJEB8624	SAMEA3389082	2005	Australia	Infection	None	A1	Human
E0732	PRJEB8624	SAMEA3389083	2007	Australia	Infection	vanB	A1	Human
E0733	PRJEB8624	SAMEA3389084	2007	Australia	Infection	None	A1	Human
E0734	PRJEB8624	SAMEA3389085	2011	Australia	Infection	vanB	A1	Human
E0735	PRJEB8624	SAMEA3389086	2011	Australia	Infection	vanB	A1	Human
E0736	PRJEB8624	SAMEA3389087	2011	Australia	Infection	None	A1	Human
E0737	PRJEB8624	SAMEA3389088	2012	Australia	Infection	None	A1	Human
E0738	PRJEB8624	SAMEA3389089	2004	Australia	Infection	None	A1	Human
E0739	PRJEB8624	SAMEA3389090	2006	Australia	Infection	None	A1	Human
E0740	PRJEB8624	SAMEA3389091	2007	Australia	Infection	vanB	A1	Human
E0741	PRJEB8624	SAMEA3389092	2007	Australia	Infection	None	A2	Human
E0742	PRJEB8624	SAMEA3389093	2011	Australia	Infection	vanB	A1	Human
E0743	PRJEB8624	SAMEA3389094	2011	Australia	Infection	None	A1	Human
E0744	PRJEB8624	SAMEA3389095	2012	Australia	Infection	None	A1	Human
E0745	PRJEB8624	SAMEA3389096	2012	Australia	Infection	vanB	A1	Human
E0746	PRJEB8624	SAMEA3389097	2006	Australia	Infection	None	A1	Human
E0747	PRJEB8624	SAMEA3389098	2007	Australia	Infection	vanB	A1	Human
E0748	PRJEB8624	SAMEA3389099	2011	Australia	Infection	None	A1	Human
E0749	PRJEB8624	SAMEA3389100	2011	Australia	Infection	vanB	A1	Human
E0750	PRJEB8624	SAMEA3389101	2011	Australia	Infection	None	A1	Human
E0751	PRJEB8624	SAMEA3389102	2011	Australia	Infection	vanB	A1	Human
E0752	PRJEB8624	SAMEA3389103	2012	Australia	Infection	None	A1	Human

E0753	PRJEB8624	SAMEA3389104	2004	Australia	Infection	None	A1	Human
E0754	PRJEB8624	SAMEA3389105	2006	Australia	Infection	None	A1	Human
E0755	PRJEB8624	SAMEA3389106	2007	Australia	Infection	None	A1	Human
E0756	PRJEB8624	SAMEA3389107	2011	Australia	Infection	None	A1	Human
E0757	PRJEB8624	SAMEA3389108	2011	Australia	Infection	vanB	A1	Human
E0758	PRJEB8624	SAMEA3389109	2011	Australia	Infection	None	A1	Human
E0759	PRJEB8624	SAMEA3389110	2012	Australia	Infection	vanB	A1	Human
E0760	PRJEB8624	SAMEA3389111	2012	Australia	Infection	None	A1	Human
E0761	PRJEB8624	SAMEA3389112	2006	Australia	Infection	None	A1	Human
E0762	PRJEB8624	SAMEA3389113	2007	Australia	Infection	vanB	A1	Human
E0763	PRJEB8624	SAMEA3389114	2011	Australia	Infection	vanB	A1	Human
E0764	PRJEB8624	SAMEA3389115	2011	Australia	Infection	vanB	A1	Human
E0765	PRJEB8624	SAMEA3389116	2011	Australia	Infection	None	A1	Human
E0766	PRJEB8624	SAMEA3389117	2012	Australia	Infection	vanB	A1	Human
E0767	PRJEB8624	SAMEA3389118	2012	Australia	Infection	None	A1	Human
E0768	PRJNA636894	SAMN15088179	2000	Brazil	Screening	None	B	Human
E0769	PRJNA636894	SAMN15088180	2000	Brazil	Screening	None	A2	Human
E0770	PRJNA636894	SAMN15088181	2001	Brazil	Screening	None	A2	Human
E0771	PRJNA636894	SAMN15088182	2001	Brazil	Screening	None	A2	Human
E0772	PRJNA636894	SAMN15088183	1997	Brazil	Infection	vanA	A2	Human
E0773	PRJNA636894	SAMN15088184	1998	Brazil	Screening	vanA	A1	Human
E0774	PRJNA636894	SAMN15088185	2001	Tanzania	Infection	None	A1	Human
E0775	PRJNA636894	SAMN15088186	2001	Tanzania	Infection	None	A2	Human
E0776	PRJNA636894	SAMN15088187	2001	Tanzania	Infection	None	A1	Human
E0777	PRJNA636894	SAMN15088188	2001	Tanzania	Infection	None	A1	Human
E0778	PRJNA636894	SAMN15088189	2001	Tanzania	Infection	None	A1	Human
E0779	PRJNA636894	SAMN15088190	2001	Tanzania	Infection	None	B	Human

E0780	PRJNA342552	SAMN05831040	2000	Australia	Infection	vanA	A1	Human
E0781	PRJNA342552	SAMN05831041	2001	Australia	Infection	None	B	Human
E0782	PRJNA342552	SAMN05831042	2005	Australia	Infection	None	A1	Human
E0783	PRJNA342552	SAMN05831043	2005	Australia	Infection	None	B	Human
E0784	PRJNA342552	SAMN05831044	2006	Australia	Infection	None	A1	Human
E0785	PRJNA342552	SAMN05831046	2011	Australia	Infection	None	A1	Human
E0786	PRJNA342552	SAMN05831047	2011	Australia	Infection	None	B	Human
E0787	PRJNA342552	SAMN05831048	2011	Australia	Infection	vanB	A1	Human
E0788	PRJNA342552	SAMN05831049	2011	Australia	Infection	None	A1	Human
E0789	PRJNA342552	SAMN05831050	2011	Australia	Infection	None	A1	Human
E0790	PRJNA342552	SAMN05831051	2011	Australia	Infection	vanB	A1	Human
E0791	PRJNA342552	SAMN05831052	2011	Australia	Infection	None	A1	Human
E0792	PRJNA342552	SAMN05831053	2011	Australia	Infection	None	A2	Human
E0793	PRJNA342552	SAMN05831054	2011	Australia	Infection	None	A1	Human
E0794	PRJNA342552	SAMN05831055	2011	Australia	Infection	None	A1	Human
E0795	PRJNA342552	SAMN05831056	2011	Australia	Infection	vanB	A1	Human
E0796	PRJNA342552	SAMN05831057	2011	Australia	Infection	None	A2	Human
E0797	PRJNA342552	SAMN05831058	2011	Australia	Infection	vanB	A1	Human
E0798	PRJNA342552	SAMN05831059	2011	Australia	Infection	vanB	A1	Human
E0799	PRJNA342552	SAMN05831060	2012	Australia	Infection	vanB	A1	Human
E0800	PRJNA342552	SAMN05831061	2012	Australia	Infection	None	A1	Human
E0801	PRJNA342552	SAMN05831062	2012	Australia	Infection	vanB	A1	Human
E0802	PRJNA342552	SAMN05831063	2012	Australia	Infection	None	B	Human
E0803	PRJNA342552	SAMN05831064	2012	Australia	Infection	vanB	A1	Human
E0804	PRJNA342552	SAMN05831065	2012	Australia	Infection	None	A2	Human
E0805	PRJNA342552	SAMN05831066	2012	Australia	Infection	None	A1	Human
E0806	PRJNA342552	SAMN05831067	2012	Australia	Infection	None	A1	Human

E0807	PRJNA342552	SAMN05831068	2012	Australia	Infection	vanB	A1	Human
E0808	PRJNA342552	SAMN05831069	2012	Australia	Infection	None	A1	Human
E0809	PRJNA342552	SAMN05831070	2012	Australia	Infection	vanB	A1	Human
E0810	PRJNA342552	SAMN05831071	2012	Australia	Infection	vanB	A1	Human
E0811	PRJNA342552	SAMN05831072	2013	Australia	Infection	None	A1	Human
E0812	PRJNA342552	SAMN05831073	2013	Australia	Infection	None	A1	Human
E0813	PRJNA342552	SAMN05831074	2013	Australia	Infection	None	A1	Human
E0814	PRJNA342552	SAMN05831075	2013	Australia	Infection	None	A1	Human
E0815	PRJNA342552	SAMN05831076	2013	Australia	Infection	vanB	A1	Human
E0816	PRJNA342552	SAMN05831077	2013	Australia	Infection	None	A2	Human
E0817	PRJNA342552	SAMN05831078	2013	Australia	Infection	None	A1	Human
E0818	PRJNA342552	SAMN05831079	2013	Australia	Infection	None	A2	Human
E0819	PRJNA342552	SAMN05831080	2013	Australia	Infection	None	B	Human
E0820	PRJNA342552	SAMN05831081	2013	Australia	Infection	vanB	A1	Human
E0821	PRJNA342552	SAMN05831082	2014	Australia	Infection	vanB	A1	Human
E0822	PRJNA342552	SAMN05831083	2014	Australia	Infection	None	A1	Human
E0823	PRJNA342552	SAMN05831084	2014	Australia	Infection	None	A1	Human
E0824	PRJNA342552	SAMN05831086	2014	Australia	Infection	None	A1	Human
E0825	PRJNA342552	SAMN05831087	2014	Australia	Infection	vanB	A1	Human
E0826	PRJNA342552	SAMN05831088	2014	Australia	Infection	None	A1	Human
E0827	PRJNA342552	SAMN05831089	2015	Australia	Infection	None	A1	Human
E0828	PRJNA342552	SAMN05831090	2015	Australia	Infection	vanB	A1	Human
E0829	PRJNA342552	SAMN05831091	2015	Australia	Infection	vanA	A1	Human
E0830	PRJNA342552	SAMN05831092	2015	Australia	Infection	vanB	A1	Human
E0831	PRJNA310320	SAMN04450365	2004	Germany	Infection	vanB	A1	Human
E0832	PRJNA310320	SAMN04450367	2004	Germany	Infection	vanA	A1	Human
E0833	PRJNA310320	SAMN04450369	2005	Germany	Infection	vanB	A1	Human

E0834	PRJNA310320	SAMN04450372	2006	Germany	Infection	None	A1	Human
E0835	PRJNA310320	SAMN04450374	2007	Germany	Infection	None	A1	Human
E0836	PRJNA310320	SAMN04450375	2007	Germany	Infection	None	A1	Human
E0837	PRJNA310320	SAMN04450377	2007	Germany	Infection	None	A1	Human
E0838	PRJNA310320	SAMN04450378	2007	Germany	Infection	None	A1	Human
E0839	PRJNA310320	SAMN04450380	2008	Germany	Infection	None	A1	Human
E0840	PRJNA310320	SAMN04450381	2008	Germany	Screening	vanB	A1	Human
E0841	PRJNA310320	SAMN04450382	2008	Germany	Screening	vanB	A1	Human
E0842	PRJNA310320	SAMN04450383	2009	Germany	Infection	vanB	A1	Human
E0843	PRJNA310320	SAMN04450384	2009	Germany	Infection	vanB	A1	Human
E0844	PRJNA310320	SAMN04450385	2009	Germany	Screening	vanB	A1	Human
E0845	PRJNA310320	SAMN04450387	2009	Germany	Infection	vanB	A1	Human
E0846	PRJNA310320	SAMN04450388	2009	Germany	Infection	vanB	A1	Human
E0847	PRJNA310320	SAMN04450389	2009	Germany	Infection	None	A1	Human
E0848	PRJNA310320	SAMN04450390	2009	Germany	Infection	vanB	A1	Human
E0849	PRJNA310320	SAMN04450391	2010	Germany	Infection	vanB	A1	Human
E0850	PRJNA310320	SAMN04450392	2010	Germany	Infection	None	A1	Human
E0851	PRJNA310320	SAMN04450393	2010	Germany	Infection	vanB	A1	Human
E0852	PRJNA310320	SAMN04450394	2010	Germany	Infection	vanB	A1	Human
E0853	PRJNA310320	SAMN04450395	2010	Germany	Infection	vanB	A1	Human
E0854	PRJNA310320	SAMN04450396	2010	Germany	Infection	vanB	A1	Human
E0855	PRJNA310320	SAMN04450397	2011	Germany	Infection	None	A1	Human
E0856	PRJNA310320	SAMN04450398	2011	Germany	Infection	vanB	A1	Human
E0857	PRJNA310320	SAMN04450401	2011	Germany	Screening	vanB	A1	Human
E0858	PRJNA310320	SAMN04450404	2011	Germany	Infection	vanB	A1	Human
E0859	PRJNA310320	SAMN04450405	2011	Germany	Infection	vanB	A1	Human
E0860	PRJNA310320	SAMN04450406	2011	Germany	Infection	vanB	A1	Human

E0861	PRJNA310320	SAMN04450359	2013	Germany	Infection	vanB	A1	Human
E0862	PRJNA310320	SAMN04450360	2013	Germany	Infection	vanB	A1	Human
E0863	PRJNA310320	SAMN04450361	2013	Germany	Infection	vanB	A1	Human
E0864	PRJNA310320	SAMN04450362	2014	Germany	Infection	vanB	A1	Human
E0865	PRJNA310320	SAMN04450363	2014	Germany	Infection	vanA	A1	Human
E0866	PRJNA310320	SAMN04450364	2004	Germany	Infection	vanB	A1	Human
E0867	PRJNA310320	SAMN04450373	2007	Germany	Infection	vanB	A1	Human
E0868	PRJNA310320	SAMN04450379	2007	Germany	Infection	vanB	A1	Human
E0869	PRJNA310320	SAMN04450376	2007	Germany	Infection	vanB	A1	Human
E0870	PRJNA310320	SAMN04450407	2012	Germany	Infection	vanB	A1	Human
E0871	PRJNA310320	SAMN04450368	2005	Germany	Infection	vanB	A1	Human
E0872	PRJNA310320	SAMN04450408	2012	Germany	Infection	vanB	A1	Human
E0873	PRJNA310320	SAMN04450366	2004	Germany	Infection	vanB	A1	Human
E0874	PRJNA310320	SAMN04450400	2011	Germany	Infection	vanB	A1	Human
E0875	PRJNA310320	SAMN04450386	2009	Germany	Infection	vanB	A1	Human
E0876	PRJNA310320	SAMN04450399	2011	Germany	Infection	vanB	A1	Human
E0877	PRJNA310320	SAMN04450402	2011	Germany	Infection	vanB	A1	Human
E0878	PRJNA310320	SAMN04450403	2011	Germany	Infection	vanB	A1	Human
E0879	PRJNA310320	SAMN04450370	2006	Germany	Infection	vanB	A1	Human
E0880	PRJNA328573	SAMN05375116	2014	Germany	Screening	vanA	A1	Human
E0881	PRJNA328573	SAMN05375117	2014	Germany	Infection	vanB	A1	Human
E0882	PRJNA328573	SAMN05375118	2014	Germany	Screening	vanA	A1	Human
E0883	PRJNA328573	SAMN05375119	2015	Germany	Screening	vanA	A1	Human
E0884	PRJNA636894	SAMN15088191	2016	Germany	Infection	vanA	A1	Human
E0885	PRJNA636894	SAMN15088192	2016	Germany	Infection	vanA	A1	Human
E0886	PRJNA636894	SAMN15088193	2016	Germany	Infection	vanB	A1	Human
E0887	PRJNA636894	SAMN15088194	2016	Germany	Infection	vanB	A1	Human

E0888	PRJNA636894	SAMN15088195	2016	Germany	Infection	vanB	A1	Human
E0889	PRJNA636894	SAMN15088196	2016	Germany	Infection	vanA	A1	Human
E0890	PRJNA636894	SAMN15088197	2016	Germany	Infection	vanA	A1	Human
E0891	PRJNA636894	SAMN15088198	2016	Germany	Infection	vanB	A1	Human
E0892	PRJNA636894	SAMN15088199	2016	Germany	Infection	vanB	A1	Human
E0893	PRJNA636894	SAMN15088200	2016	Germany	Infection	vanB	A1	Human
E0894	PRJNA636894	SAMN15088201	2016	Germany	Infection	vanB	A1	Human
E0895	PRJNA636894	SAMN15088202	2016	Germany	Infection	vanA	A1	Human
E0896	PRJNA636894	SAMN15088203	2016	Germany	Infection	vanB	A1	Human
E0897	PRJNA636894	SAMN15088204	2016	Germany	Infection	vanB	A1	Human
E0898	PRJNA636894	SAMN15088205	2016	Germany	Infection	None	A2	Human
E0899	PRJNA636894	SAMN15088206	2016	Germany	Infection	vanB	A1	Human
E0900	PRJNA636894	SAMN15088207	2016	Germany	Infection	vanB	A1	Human
E0901	PRJNA636894	SAMN15088208	2016	Germany	Infection	vanB	A1	Human
E0902	PRJNA636894	SAMN15088209	2016	Germany	Infection	vanB	A1	Human
E0903	PRJNA636894	SAMN15088210	2016	Germany	Infection	vanB	A1	Human
E0904	PRJNA636894	SAMN15088211	2016	Germany	Infection	vanB	A1	Human
E0905	PRJNA636894	SAMN15088212	2016	Germany	Infection	None	A1	Human
E0906	PRJNA636894	SAMN15088213	2016	Germany	Infection	None	A1	Human
E0907	PRJNA636894	SAMN15088214	2016	Germany	Infection	None	A1	Human
E0908	PRJNA636894	SAMN15088215	2016	Germany	Infection	vanB	A1	Human
E0909	PRJNA636894	SAMN15088216	2016	Germany	Infection	vanB	A1	Human
E0910	PRJNA636894	SAMN15088217	2016	Germany	Infection	vanA	A1	Human
E0911	PRJNA636894	SAMN15088218	2016	Germany	Infection	vanA	A1	Human
E0912	PRJNA636894	SAMN15088219	2016	Germany	Infection	vanA	A1	Human
E0913	PRJNA636894	SAMN15088220	2016	Germany	Screening	vanA	A1	Human
E0914	PRJNA636894	SAMN15088221	2016	Germany	Infection	vanA	A1	Human

E0915	PRJNA636894	SAMN15088222	2016	Germany	Infection	vanB	A1	Human
E0916	PRJNA636894	SAMN15088223	2016	Germany	Infection	None	A1	Human
E0917	PRJNA636894	SAMN15088224	2016	Germany	Infection	vanB	A1	Human
E0918	PRJNA636894	SAMN15088225	2016	Germany	Infection	vanB	A1	Human
E0919	PRJNA636894	SAMN15088226	2016	Germany	Infection	vanA	A1	Human
E0920	PRJNA636894	SAMN15088227	2016	Germany	Infection	vanB	A1	Human
E0921	PRJNA636894	SAMN15088228	2016	Germany	Infection	vanB	A1	Human
E0922	PRJNA636894	SAMN15088229	2016	Germany	Infection	vanA	A1	Human
E0923	PRJNA636894	SAMN15088230	2016	Germany	Infection	vanB	A1	Human
E0924	PRJNA636894	SAMN15088231	2016	Germany	Infection	vanB	A1	Human
E0925	PRJNA636894	SAMN15088232	2016	Germany	Infection	vanB	A1	Human
E0926	PRJNA636894	SAMN15088233	2016	Germany	Infection	vanB	A1	Human
E0927	PRJNA636894	SAMN15088234	2016	Germany	Infection	vanB	A1	Human
E0928	PRJNA636894	SAMN15088235	2016	Germany	Screening	vanA	A1	Human
E0929	PRJNA636894	SAMN15088236	2016	Germany	Infection	vanC	A1	Human
E0930	PRJNA636894	SAMN15088237	2016	Germany	Infection	vanB	A1	Human
E0931	PRJNA636894	SAMN15088238	2016	Germany	Infection	vanA	A1	Human
E0932	PRJNA636894	SAMN15088239	2016	Germany	Infection	None	A1	Human
E0933	PRJNA636894	SAMN15088240	2016	Germany	Infection	vanB	A1	Human
E0934	PRJNA636894	SAMN15088241	2016	Germany	Infection	vanB	A1	Human
E0935	PRJNA636894	SAMN15088242	2016	Germany	Infection	vanB	A1	Human
E0936	PRJNA636894	SAMN15088243	2016	Germany	Infection	vanB	A1	Human
E0937	PRJNA636894	SAMN15088244	2016	Germany	Infection	None	A2	Human
E0938	PRJNA636894	SAMN15088245	2016	Germany	Infection	None	B	Human
E0939	PRJNA636894	SAMN15088246	2016	Germany	Infection	None	A1	Human
E0940	PRJNA636894	SAMN15088247	2016	Germany	Infection	vanB	A1	Human
E0941	PRJNA636894	SAMN15088248	2016	Germany	Infection	vanB	A1	Human

E0942	PRJNA636894	SAMN15088249	2016	Germany	Infection	vanB	A1	Human
E0943	PRJNA636894	SAMN15088250	2016	Germany	Infection	None	A1	Human
E0944	PRJNA636894	SAMN15088251	2014	Japan	Infection	None	A1	Human
E0945	PRJNA636894	SAMN15088252	2014	Japan	Infection	None	B	Human
E0946	PRJNA636894	SAMN15088253	2014	Japan	Infection	None	A2	Human
E0947	PRJNA636894	SAMN15088254	2014	Japan	Infection	None	A1	Human
E0948	PRJNA636894	SAMN15088255	2014	Japan	Screening	None	A1	Human
E0949	PRJNA636894	SAMN15088256	2014	Japan	Infection	None	A1	Human
E0950	PRJNA636894	SAMN15088257	2014	Japan	Infection	None	B	Human
E0951	PRJNA636894	SAMN15088258	2014	Japan	Infection	None	A1	Human
E0952	PRJNA636894	SAMN15088259	2014	Japan	Infection	None	A1	Human
E0953	PRJNA636894	SAMN15088260	2014	Japan	Infection	None	A1	Human
E0954	PRJNA636894	SAMN15088261	2014	Japan	Infection	None	A1	Human
E0955	PRJNA636894	SAMN15088262	2014	Japan	Infection	None	A1	Human
E0956	PRJNA636894	SAMN15088263	2014	Japan	Infection	None	A1	Human
E0957	PRJNA636894	SAMN15088264	2014	Japan	Infection	None	A2	Human
E0958	PRJNA636894	SAMN15088265	2014	Japan	Infection	None	A1	Human
E0959	PRJNA636894	SAMN15088266	2014	Japan	Screening	None	A1	Human
E0960	PRJNA636894	SAMN15088267	2014	Japan	Infection	None	A1	Human
E0961	PRJNA636894	SAMN15088268	2014	Japan	Infection	None	A1	Human
E0962	PRJNA636894	SAMN15088269	2014	Japan	Infection	None	A1	Human
E0963	PRJNA636894	SAMN15088270	2014	Japan	Infection	None	A1	Human
E0964	PRJNA636894	SAMN15088271	2014	Japan	Infection	None	A1	Human
E0965	PRJNA636894	SAMN15088272	2014	Japan	Infection	None	A1	Human
E0966	PRJNA636894	SAMN15088273	2014	Japan	Infection	None	A1	Human
E0967	PRJNA636894	SAMN15088274	2014	Japan	Infection	None	B	Human
E0968	PRJNA636894	SAMN15088275	2014	Japan	Infection	None	A1	Human

E0969	PRJNA636894	SAMN15088276	2014	Japan	Infection	None	A1	Human
E0970	PRJNA636894	SAMN15088277	2014	Japan	Infection	None	A1	Human
E0971	PRJNA636894	SAMN15088278	2014	Japan	Infection	None	A1	Human
E0972	PRJNA636894	SAMN15088279	2014	Japan	Infection	None	A1	Human
E0973	PRJNA636894	SAMN15088280	2014	Japan	Infection	None	A1	Human
E0974	PRJNA636894	SAMN15088281	2014	Japan	Infection	None	A1	Human
E0975	PRJNA636894	SAMN15088282	2014	Japan	Infection	None	B	Human
E0976	PRJNA636894	SAMN15088283	2014	Japan	Infection	None	A1	Human
E0977	PRJNA636894	SAMN15088284	2014	Japan	Infection	None	A1	Human
E0978	PRJNA636894	SAMN15088285	2014	Japan	Infection	None	B	Human
E0979	PRJNA636894	SAMN15088286	2014	Japan	Infection	None	A1	Human
E0980	PRJNA636894	SAMN15088287	2014	Japan	Infection	None	A1	Human
E0981	PRJNA636894	SAMN15088288	2014	Japan	Screening	None	A1	Human
E0982	PRJNA636894	SAMN15088289	2014	Japan	Infection	None	A1	Human
E0983	PRJNA636894	SAMN15088290	2014	Japan	Infection	None	A1	Human
E0984	PRJNA636894	SAMN15088291	2014	Japan	Infection	None	A1	Human
E0985	PRJNA636894	SAMN15088292	2014	Japan	Infection	None	A1	Human
E0986	PRJNA636894	SAMN15088293	2014	Japan	Infection	None	A1	Human
E0987	PRJNA636894	SAMN15088294	2014	Japan	Infection	None	A1	Human
E0988	PRJNA636894	SAMN15088295	2014	Japan	Infection	None	A1	Human
E0989	PRJNA636894	SAMN15088296	2014	Japan	Infection	None	A1	Human
E0990	PRJNA636894	SAMN15088297	2014	Japan	Infection	None	A1	Human
E0991	PRJNA636894	SAMN15088298	2014	Japan	Infection	None	A1	Human
E0992	PRJNA636894	SAMN15088299	2014	Japan	Infection	None	A1	Human
E0993	PRJNA636894	SAMN15088300	2014	Japan	Infection	None	A1	Human
E0994	PRJNA636894	SAMN15088301	2014	Japan	Infection	None	A1	Human
E0995	PRJNA636894	SAMN15088302	2014	Japan	Screening	None	A1	Human

E0996	PRJNA636894	SAMN15088303	2014	Japan	Infection	None	A1	Human
E0997	PRJNA636894	SAMN15088304	2014	Japan	Infection	None	A2	Human
E0998	PRJNA636894	SAMN15088305	2014	Japan	Infection	None	A1	Human
E0999	PRJNA636894	SAMN15088306	2014	Japan	Infection	None	A1	Human
E1000	PRJNA636894	SAMN15088307	2014	Japan	Infection	None	A1	Human
E1001	PRJNA636894	SAMN15088308	2014	Japan	Infection	None	A1	Human
E1002	PRJNA636894	SAMN15088309	2014	Japan	Infection	None	A1	Human
E1003	PRJNA636894	SAMN15088310	2014	Japan	Infection	None	A1	Human
E1004	PRJNA636894	SAMN15088311	2014	Japan	Infection	None	A1	Human
E1005	PRJNA636894	SAMN15088312	2014	Japan	Infection	None	B	Human
E1006	PRJNA636894	SAMN15088313	2014	Japan	Infection	None	A2	Human
E1007	PRJNA636894	SAMN15088314	2014	Japan	Infection	None	A1	Human
E1008	PRJNA636894	SAMN15088315	2014	Japan	Infection	None	A1	Human
E1009	PRJNA636894	SAMN15088316	2014	Japan	Infection	None	A1	Human
E1010	PRJNA636894	SAMN15088317	2014	Japan	Infection	None	A1	Human
E1011	PRJNA636894	SAMN15088318	2014	Japan	Infection	None	A1	Human
E1012	PRJNA636894	SAMN15088319	2014	Japan	Screening	None	A1	Human
E1013	PRJNA636894	SAMN15088320	2014	Japan	Infection	None	A1	Human
E1014	PRJNA636894	SAMN15088321	2015	Japan	Infection	None	A1	Human
E1015	PRJNA636894	SAMN15088322	2015	Japan	Infection	None	A1	Human
E1016	PRJNA636894	SAMN15088323	2015	Japan	Infection	None	B	Human
E1017	PRJNA636894	SAMN15088324	2015	Japan	Infection	None	A1	Human
E1018	PRJNA636894	SAMN15088325	2015	Japan	Infection	None	A1	Human
E1019	PRJNA636894	SAMN15088326	2015	Japan	Infection	None	B	Human
E1020	PRJNA636894	SAMN15088327	2015	Japan	Infection	None	A1	Human
E1021	PRJNA636894	SAMN15088328	2015	Japan	Infection	None	A1	Human
E1022	PRJNA636894	SAMN15088329	2015	Japan	Infection	None	A1	Human

E1023	PRJNA636894	SAMN15088330	2015	Japan	Screening	None	A1	Human
E1024	PRJNA636894	SAMN15088331	2015	Japan	Infection	None	A1	Human
E1025	PRJNA636894	SAMN15088332	2015	Japan	Infection	None	A1	Human
E1026	PRJNA636894	SAMN15088333	2015	Japan	Infection	None	A1	Human
E1027	PRJNA636894	SAMN15088334	2015	Japan	Screening	None	A1	Human
E1028	PRJNA636894	SAMN15088335	2015	Japan	Infection	None	A1	Human
E1029	PRJNA636894	SAMN15088336	2015	Japan	Infection	None	A1	Human
E1030	PRJNA636894	SAMN15088337	2015	Japan	Infection	None	A1	Human
E1031	PRJNA636894	SAMN15088338	2015	Japan	Infection	None	B	Human
E1032	PRJNA636894	SAMN15088339	2015	Japan	Infection	None	A1	Human
E1033	PRJNA636894	SAMN15088340	2015	Japan	Screening	None	A2	Human
E1034	PRJNA636894	SAMN15088341	2015	Japan	Infection	None	A1	Human
E1035	PRJNA636894	SAMN15088342	2015	Japan	Screening	None	A1	Human
E1036	PRJNA636894	SAMN15088343	2015	Japan	Infection	None	A2	Human
E1037	PRJNA636894	SAMN15088344	2015	Japan	Screening	None	A1	Human
E1038	PRJNA636894	SAMN15088345	2015	Japan	Infection	None	A1	Human
E1039	PRJNA636894	SAMN15088346	2015	Japan	Infection	None	A1	Human
E1040	PRJNA636894	SAMN15088347	2011	Belgium	Infection	None	A1	Human
E1041	PRJNA636894	SAMN15088348	2012	Belgium	Infection	None	A1	Human
E1042	PRJNA552025	SAMN12640957	2011	Belgium	Infection	None	A1	Human
E1043	PRJNA552025	SAMN13164758	2016	Belgium	Infection	None	A1	Human
E1044	PRJNA552025	SAMN13164759	2016	Belgium	Infection	None	A1	Human
E1045	PRJNA552025	SAMN13157963	2012	Belgium	Infection	None	A1	Human
E1046	PRJNA552025	SAMN13195566	2012	Belgium	Infection	None	A1	Human
E1047	PRJNA552025	SAMN13155795	2012	Belgium	Infection	None	A1	Human
E1048	PRJNA552025	SAMN13159150	2014	Belgium	Infection	None	A1	Human
E1049	PRJNA552025	SAMN12640948	2015	Belgium	Infection	None	A1	Human

E1050	PRJNA552025	SAMN13159147	2014	Belgium	Infection	None	A1	Human
E1051	PRJNA552025	SAMN13159157	2014	Belgium	Infection	None	A1	Human
E1052	PRJNA552025	SAMN13159158	2014	Belgium	Infection	None	A1	Human
E1053	PRJNA552025	SAMN13164754	2015	Belgium	Infection	None	A1	Human
E1054	PRJNA552025	SAMN13164752	2015	Belgium	Infection	None	A1	Human
E1055	PRJNA552025	SAMN13195567	2015	Belgium	Infection	None	A1	Human
E1056	PRJNA552025	SAMN13159152	2014	Belgium	Infection	None	A1	Human
E1057	PRJNA636894	SAMN15088349	2014	Belgium	Infection	None	A1	Human
E1058	PRJNA552025	SAMN13159160	2014	Belgium	Infection	None	A1	Human
E1059	PRJNA552025	SAMN13159144	2013	Belgium	Infection	None	A1	Human
E1060	PRJNA552025	SAMN13159151	2014	Belgium	Infection	None	A1	Human
E1061	PRJNA552025	SAMN13159142	2013	Belgium	Infection	None	A1	Human
E1062	PRJNA552025	SAMN13159159	2014	Belgium	Infection	None	A1	Human
E1063	PRJNA552025	SAMN13164791	2015	Belgium	Infection	None	A1	Human
E1064	PRJNA552025	SAMN13159155	2014	Belgium	Infection	None	A1	Human
E1065	PRJNA552025	SAMN13164748	2015	Belgium	Infection	None	A1	Human
E1066	PRJNA552025	SAMN13164751	2015	Belgium	Infection	None	A1	Human
E1067	PRJNA552025	SAMN13164757	2015	Belgium	Infection	None	A1	Human
E1068	PRJNA552025	SAMN13164756	2015	Belgium	Infection	None	A1	Human
E1069	PRJNA552025	SAMN12640949	2015	Belgium	Infection	None	A1	Human
E1070	PRJNA552025	SAMN13159148	2014	Belgium	Infection	None	A1	Human
E1071	PRJNA552025	SAMN13159149	2014	Belgium	Infection	None	A1	Human
E1072	PRJNA552025	SAMN13164750	2015	Belgium	Infection	None	A1	Human
E1073	PRJNA342552	SAMN05831045	2011	Australia	Infection	None	A1	Human
E1074	PRJNA342552	SAMN05831085	2014	Australia	Infection	None	A2	Human
E1075	PRJNA636894	SAMN15088350	Unknown	Brazil	Screening	None	A2	Human
E1076	PRJNA552025	SAMN12640964	2013	Belgium	Infection	vanA	A2	Human

E1077	PRJNA552025	SAMN12640969	2004	Belgium	Infection	vanA	A1	Human
E1078	PRJNA552025	SAMN12634763	2009	Belgium	Infection	vanB	A1	Human
E1079	PRJNA552025	SAMN13157960	2012	Belgium	Infection	vanA	A1	Human
E1080	PRJNA552025	SAMN12634766	2012	Belgium	Infection	vanA	A1	Human
E1081	PRJNA552025	SAMN13155793	2012	Belgium	Infection	vanB	A1	Human
E1082	PRJNA552025	SAMN13155791	2009	Belgium	Infection	vanB	A1	Human
E1083	PRJNA552025	SAMN13157962	2012	Belgium	Infection	vanA	A1	Human
E1084	PRJNA552025	SAMN13164767	2016	Belgium	Infection	vanA	A1	Human
E1085	PRJNA552025	SAMN13164770	2016	Belgium	Infection	vanA	A1	Human
E1086	PRJNA552025	SAMN13164772	2016	Belgium	Infection	vanA	A1	Human
E1087	PRJNA552025	SAMN12640966	2016	Belgium	Infection	vanA	A1	Human
E1088	PRJNA552025	SAMN13164773	2016	Belgium	Infection	vanA	A1	Human
E1089	PRJNA552025	SAMN13155790	2008	Belgium	Infection	vanB	A1	Human
E1090	PRJNA552025	SAMN13168598	2002	Belgium	Infection	vanB	A1	Human
E1091	PRJNA552025	SAMN12640968	2003	Belgium	Infection	vanA	A1	Human
E1092	PRJNA552025	SAMN12634760	2003	Belgium	Infection	vanA	A1	Human
E1093	PRJNA552025	SAMN12634761	2007	Belgium	Infection	vanA	A1	Human
E1094	PRJNA552025	SAMN13164786	2012	Belgium	Infection	vanA	A1	Human
E1095	PRJNA552025	SAMN12640959	2012	Belgium	Infection	vanA	A1	Human
E1096	PRJNA636894	SAMN15088351	2007	Belgium	Infection	vanA	A1	Human
E1097	PRJNA552025	SAMN13168603	2005	Belgium	Infection	vanA	A1	Human
E1098	PRJNA552025	SAMN12634762	2009	Belgium	Infection	None	A1	Human
E1099	PRJNA636894	SAMN15088352	2009	Belgium	Infection	vanA	A1	Human
E1100	PRJNA552025	SAMN13155792	2011	Belgium	Infection	vanB	A1	Human
E1101	PRJNA636894	SAMN15088353	2007	Belgium	Infection	vanB	A1	Human
E1102	PRJNA552025	SAMN13155794	2011	Belgium	Infection	vanB	A1	Human
E1103	PRJNA552025	SAMN12634767	2011	Belgium	Infection	vanA	A1	Human

E1104	PRJNA552025	SAMN13157961	2010	Belgium	Infection	vanB	A1	Human
E1105	PRJNA552025	SAMN12634764	2010	Belgium	Infection	vanA	A1	Human
E1106	PRJNA552025	SAMN12640958	2011	Belgium	Infection	vanB	A1	Human
E1107	PRJNA552025	SAMN13155821	2011	Belgium	Infection	vanA	A1	Human
E1108	PRJNA552025	SAMN12560306	2008	Belgium	Infection	vanA	A1	Human
E1109	PRJNA552025	SAMN13195565	2010	Belgium	Infection	None	A1	Human
LB01	PRJNA73043	SAMN00779812	2001	Ireland	Screening	None	B	Human
LB02	PRJNA73043	SAMN00779819	2000	Netherlands	Infection	None	B	Human
LB03	PRJNA73043	SAMN00779813	2004	Netherlands	Infection	None	B	Human
LB04	PRJNA73043	SAMN00779801	2006	Netherlands	Infection	None	B	Human
LB05	PRJNA73043	SAMN00779820	2001	Spain	Screening	None	B	Human
LB06	PRJNA73043	SAMN00779808	2000	Germany	Infection	None	B	Human
LB07	PRJNA73043	SAMN00779818	1998	Netherlands	Screening	None	B	Human
LB08	PRJNA73043	SAMN00779803	1956	Norway	Unknown	None	B	Non-human
LB09	PRJNA73043	SAMN00779822	1964	Norway	Unknown	None	B	Non-human
LB10	PRJNA73043	SAMN00779845	2000	Germany	Unknown	None	B	Human
LA201	PRJNA73043	SAMN00779800	2001	South Africa	Unknown	None	A2	Non-human
LA202	PRJNA73043	SAMN00779833	1960	Netherlands	Infection	None	A2	Human
LA203	PRJNA73043	SAMN00779834	2001	Germany	Screening	None	A2	Non-human
LA204	PRJNA73043	SAMN00779817	1998	Netherlands	Screening	None	A2	Human
LA205	PRJNA73043	SAMN00779810	1965	Netherlands	Screening	None	A2	Human
LA206	PRJNA73043	SAMN00779829	1994	Belgium	Unknown	None	A2	Non-human
LA207	PRJNA73043	SAMN00779809	1959	Netherlands	Unknown	None	A2	Non-human
LA208	PRJNA73043	SAMN00779811	1998	France	Infection	None	A2	Human
LA209	PRJNA73043	SAMN00779831	1996	Netherlands	Screening	vanA	A2	Non-human
LA210	PRJNA73043	SAMN00779804	2004	Sweden	Unknown	vanA	A2	Non-human
LA211	PRJNA73043	SAMN00779840	1992	United Kingdom	Screening	None	A2	Non-human

LA212	PRJNA73043	SAMN00779823	1996	Netherlands	Screening	vanA	A2	Non-human
LA213	PRJNA73043	SAMN00779866	2004	Netherlands	Unknown	None	A2	Non-human
LA214	PRJNA73043	SAMN00779824	1957	Netherlands	Infection	None	A2	Human
LA215	PRJNA73043	SAMN00779867	1995	Belgium	Unknown	None	A2	Non-human
LA216	PRJNA73043	SAMN00779807	2001	Netherlands	Screening	None	A2	Human
LA217	PRJNA73043	SAMN00779855	1995	Belgium	Unknown	vanA	A2	Non-human
LA218	PRJNA73043	SAMN00779821	1995	Netherlands	Infection	vanA	A2	Human
LA219	PRJNA73043	SAMN00779814	2001	Denmark	Unknown	None	A2	Non-human
LA220	PRJNA73043	SAMN00779839	1998	Spain	Infection	None	A2	Human
LA221	PRJNA73043	SAMN00779841	2002	Netherlands	Infection	None	A2	Human
LA222	PRJNA73043	SAMN00779836	1981	Netherlands	Unknown	None	A2	Non-human
LA223	PRJNA73043	SAMN00779828	1995	Germany	Unknown	None	A2	Non-human
LA224	PRJNA73043	SAMN00779830	1982	Netherlands	Unknown	None	A2	Non-human
LA225	PRJNA73043	SAMN00779868	1995	Spain	Unknown	None	A2	Non-human
LA226	PRJNA73043	SAMN00779815	2002	Netherlands	Screening	None	A2	Human
LA227	PRJNA73043	SAMN00779816	1995	Belgium	Unknown	None	A2	Non-human
LA228	PRJNA73043	SAMN00779831	1979	Netherlands	Screening	None	A2	Human

[1]The year of *E. faecium* isolation as indicated by participating collaborators. Sequences with unknown dates were excluded from the data if time was an analysis variable.
[2] The country from which the *E. faecium* isolate was obtained
[3] Origin of *E. faecium* isolate, screening or infection isolate.
[4] Clustering category using hBAPs relative to membership of sequences from project number PRJNA73043 and labelled (LA201-LA228, LB01-LB10). Clustering analysis was performed with and without the inclusion of non-human E. faecium sequences with concordant results (see text for details).
[5] Vancomycin resistance gene presence (specified by type *vanA* or *vanB*) or absence.
[6] Host of the *E. faecium* isolate. Non-human E. faecium sequences were excluded from subsequent analysis see text for details.

Supplementary Table 2: Gene proportions by hBAPs group

Gene	Non.unique.Gene.name	Annotation	No. in A1	No. in A2	No. in B
group_7989		hypothetical protein	20	60	38
group_8484		hypothetical protein	19	60	38
group_6269		hypothetical protein	19	59	38
group_6268		hypothetical protein	19	56	38
hchA_2_hchA_1	hchA_2;hchA_1	Protein/nucleic acid deglycase 1	19	56	38
group_5887		HTH-type transcriptional regulator	18	55	38
epsH_2_epsJ_2_epsJ_epsJ_1	epsH_2;epsJ_2;epsJ;epsJ_1	Putative glycosyltransferase EpsH;putative glycosyltransferase EpsJ	28	58	19
group_5508		hypothetical protein	21	59	30
group_8249		hypothetical protein	38	55	14
ptcA	ptcA	Putrescine carbamoyltransferase	29	53	17
gadC_4_aguA	gadC_4;aguA	putative glutamate/gamma-aminobutyrate antiporter;Putative agmatine deiminase	29	53	17
group_5212		hypothetical protein	22	63	9
immA	;immA	hypothetical protein;Metallopeptidase ImmA	22	62	9
group_6160		hypothetical protein	22	63	8
group_8235		hypothetical protein	19	53	17
group_5211		hypothetical protein	22	62	7
group_6728		hypothetical protein	23	51	7
group_5618		hypothetical protein	24	51	0
group_3787		hypothetical protein	24	50	0
group_7955		hypothetical protein	18	53	3
group_99		hypothetical protein	11	57	0
group_4206		hypothetical protein	18	50	0
group_5888		hypothetical protein	16	49	0

group_8469		hypothetical protein	28	39	30
fetA	fetA	putative iron export ATP-binding protein FetA	28	39	30
fetB	fetB	putative iron export permease protein FetB	28	39	30
oatA_2	oatA_2	O-acetyltransferase OatA	46	25	20
group_6325		hypothetical protein	45	22	20
group_4213		hypothetical protein	43	6	37
group_1415		hypothetical protein	43	6	37
group_4573		hypothetical protein	9	31	38
group_6956		hypothetical protein	34	21	20
clpC_2	clpC_2	putative ATP-dependent Clp protease ATP-binding subunit	17	27	30
lexA_2_lexA_1	lexA_2;lexA_1	LexA repressor	22	27	24
group_2425		hypothetical protein	6	27	38
group_1735		hypothetical protein	14	26	28
group_3167		hypothetical protein	20	28	23
group_2700		hypothetical protein	6	26	38
topB_4	topB_4	DNA topoisomerase 3	21	28	20
group_3663		hypothetical protein	21	28	20
group_3566		hypothetical protein	20	28	22
group_2235		hypothetical protein	20	28	22
group_6439	dexB	Glucan 16-alpha-glucosidase	12	21	37
group_2623		hypothetical protein	20	28	20
group_2618		hypothetical protein	20	28	21
group_1925		hypothetical protein	20	28	20
group_4522	treP_1	PTS system trehalose-specific EIIBC component	12	20	37
metE_1	metE_1	5-methyltetrahydropteroyltriglutamate--homocysteine methyltransferase	6	24	37
group_6200		hypothetical protein	4	22	37
group_3269		putative ABC transporter ATP-binding protein	3	21	38

group_3171	polC_3	DNA polymerase III PolC-type	18	22	24
gmuE_1	gmuE_1	Putative fructokinase	11	14	37
group_1139	purR_1	HTH-type transcriptional repressor PurR	11	14	37
scrB_1	scrB_1	Sucrose-6-phosphate hydrolase	11	14	37
xre_1_xre_immR	xre_1;xre;immR	HTH-type transcriptional regulator Xre;HTH-type transcriptional regulator ImmR	5	20	36
mapP_2	mapP_2	Maltose 6'-phosphate phosphatase	17	18	23
ptsG_3	ptsG_3	PTS system glucose-specific EIICBA component	17	18	23
group_4525		putative ABC transporter ATP-binding protein	3	17	38
cmtB	cmtB	Mannitol-specific cryptic phosphotransferase enzyme IIA component	15	8	34
malP_3	malP_3	Maltose phosphorylase	16	16	23
mro_3	mro_3	Aldose 1-epimerase	16	16	23
pgmB_2	pgmB_2	Beta-phosphoglucomutase	16	16	23
group_5882	manR_2;	Transcriptional regulator ManR;hypothetical protein	15	7	34
group_7502	;phnN	hypothetical protein;Ribose 15-bisphosphate phosphokinase PhnN	16	3	38
group_7993	cspLA_2	Cold shock-like protein CspLA	6	9	38
group_4994		hypothetical protein	5	14	36
group_3283		hypothetical protein	6	11	35
ulaB_1_ulaB_2	ulaB_1;ulaB_2	Ascorbate-specific PTS system EIIB component	11	8	34
group_6739		hypothetical protein	2	13	38
group_5588		Putative membrane protein insertion efficiency factor	10	6	37
ulaA_2_ulaA_1	ulaA_2;ulaA_1	Ascorbate-specific PTS system EIIC component	9	8	34
group_696		hypothetical protein	9	7	34
group_7198	xre_2;	HTH-type transcriptional regulator Xre;hypothetical protein	5	9	37
group_6526		Phosphorylated carbohydrates phosphatase	3	7	38
group_5826	yxlF	putative ABC transporter ATP-binding protein YxlF	5	9	37
manX_4_manX_1	manX_4;manX_1	PTS system mannose-specific EIIAB component	3	7	38
dgaR_1_dgaR_3	dgaR_1;dgaR_3	Transcriptional regulatory protein DagR	3	7	38

group_4339	sorA_3	PTS system sorbose-specific EIIC component	3	7	38
group_2906	frlB_1	Fructosamine deglycase FrlB	3	7	38
levD	levD	PTS system fructose-specific EIIA component	3	7	38
group_1106		hypothetical protein	5	9	37
manZ_3	manZ_3	PTS system mannose-specific EIID component	3	7	38
group_988		hypothetical protein	5	9	37
group_7292	yvbK	putative N-acetyltransferase YvbK	6	6	37
nikB	nikB	Nickel transport system permease protein NikB	1	9	38
group_2184	oppD_3	Oligopeptide transport ATP-binding protein OppD	1	9	38
oppC	oppC	Oligopeptide transport system permease protein OppC	1	9	38
amiA_sarA	amiA;sarA	Oligopeptide-binding protein AmiA;Oligopeptide-binding protein SarA	1	8	38
group_5553	nudC	NADH pyrophosphatase	11	1	37
group_4438		hypothetical protein	12	14	23
oppF_3	oppF_3	Oligopeptide transport ATP-binding protein OppF	1	8	38
sipV	sipV	Signal peptidase I V	2	8	38
thlA	thlA	Acetyl-CoA acetyltransferase	4	6	38
plc__plcA	plc;;plcA	1-phosphatidylinositol phosphodiesterase	4	5	38
group_5397		hypothetical protein	9	1	37
group_4611		hypothetical protein	9	1	37
group_3898	celA_3	PTS system cellobiose-specific EIIB component	5	6	37
group_1336		hypothetical protein	12	14	21
ywqD_2	ywqD_2	Tyrosine-protein kinase YwqD	4	4	38
group_7848		hypothetical protein	7	12	24
mngA_mngA_1	mngA;mngA_1	PTS system 2-O-alpha-mannosyl-D-glycerate-specific EIIABC component	7	12	24
manP_5_fba_3_fba_1	manP_5;fba_3;fba_1;	PTS system mannose-specific EIIBCA component;Fructose-bisphosphate aldolase;hypothetical protein	7	12	24
pepD	pepD	Dipeptidase	3	6	37

group_6224		hypothetical protein	7	2	37
abgT_2	abgT_2	p-aminobenzoyl-glutamate transport protein	3	4	38
pepV_1_pepV_2	pepV_1;pepV_2	Beta-Ala-Xaa dipeptidase	3	4	38
kdgK_2	kdgK_2	2-dehydro-3-deoxygluconokinase	7	12	24
dgaE	dgaE	D-glucosaminate-6-phosphate ammonia lyase	7	12	24
hit_2_hit_1	hit_2;hit_1	Protein hit	1	5	38
dgaF	dgaF	2-dehydro-3-deoxy-phosphogluconate aldolase	7	12	24
group_3718	fieF	Ferrous-iron efflux pump FieF	3	4	38
rnhA	rnhA	14.7 kDa ribonuclease H-like protein	3	4	38
group_7309		hypothetical protein	7	1	37
group_7137	wbbL	N-acetylglucosaminyl-diphospho-decaprenol L-rhamnosyltransferase;Rhamnosyltransferase WbbL	2	4	38
group_6650		Deacetylase	6	12	24
group_6649	licC_4	Lichenan permease IIC component	6	12	24
group_5152		hypothetical protein	7	1	37
group_4935		hypothetical protein	6	12	24
group_6290	kstR2	HTH-type transcriptional repressor KstR2	3	4	34
chiA1	chiA1	Chitinase A1	3	4	34
group_4493		hypothetical protein	6	5	33
bsr	bsr	Blasticidin-S deaminase	3	3	38
group_1217		hypothetical protein	3	3	38
group_895		hypothetical protein	8	14	23
group_6023		hypothetical protein	3	2	38
group_5834		hypothetical protein	1	3	38
group_5105		hypothetical protein	3	2	38
group_5027		hypothetical protein	1	3	38
group_4381	ybbH_2	putative HTH-type transcriptional regulator YbbH	3	2	38
group_4171		hypothetical protein	2	5	35

group_3374		hypothetical protein	3	2	38
group_3373	malX	PTS system maltose-specific EIICB component	3	2	38
group_2345	licC_6	Lichenan permease IIC component	3	2	38
srrB	srrB	Sensor protein SrrB	1	3	38
group_1045		hypothetical protein	1	3	38
group_1039		hypothetical protein	1	4	38
arlR_1	arlR_1	Response regulator ArlR	1	3	38
malY	malY	Protein MalY	3	2	38
group_6689	xerC_3;	Tyrosine recombinase XerC;hypothetical protein	3	3	36
group_5324		hypothetical protein	1	16	23
purL	purL	Phosphoribosylformylglycinamidine synthase subunit PurL	3	1	38
ptsG_2_ptsG_1	ptsG_2;ptsG_1;	PTS system glucose-specific EIICBA component;hypothetical protein	3	1	37
group_6648		hypothetical protein	1	3	36
group_6220		hypothetical protein	1	3	36
uvrA_1_uvrA_2	uvrA_1;uvrA_2	UvrABC system protein A	1	2	38
gmuC_1_gmuC_3	gmuC_1;gmuC_3	PTS system oligo-beta-mannoside-specific EIIC component	1	2	38
cysA	cysA;	Sulfate/thiosulfate import ATP-binding protein CysA;hypothetical protein	1	2	38
alsE	alsE	D-allulose-6-phosphate 3-epimerase	1	2	38
group_4546		hypothetical protein	1	2	38
group_4508		Muramidase-2	1	2	38
group_4456		hypothetical protein	1	2	38
ppm1_1	ppm1_1	Polyprenol monophosphomannose synthase	1	3	36
murJ_3	murJ_3	Lipid II flippase MurJ	1	1	39
group_2319		hypothetical protein	1	1	39
group_1399	fruA_4	PTS system fructose-specific EIIB'BC component	1	2	38
group_1332		hypothetical protein	2	1	38
group_1113	araC_1	Arabinose operon regulatory protein	1	2	38

group_948		hypothetical protein	6	12	20
manP_1	manP_1	PTS system mannose-specific EIIBCA component	1	2	38
czrA_2	;czrA_2	hypothetical protein;HTH-type transcriptional repressor CzrA	1	2	37
group_7637		Putative trans-acting regulator	1	1	38
group_7397		hypothetical protein	2	1	37
group_7368		hypothetical protein	1	1	38
group_7163		hypothetical protein	1	1	38
group_6780		hypothetical protein	1	1	38
agrA_2__agrA_3	agrA_2;;agrA_3	Accessory gene regulator A;hypothetical protein	1	1	38
group_6341		Putative trans-acting regulator;hypothetical protein	1	1	38
nox_3_nox_2	nox_3;nox_2	NADH oxidase	3	5	29
rluB_2	rluB_2	Ribosomal large subunit pseudouridine synthase B	1	1	38
group_6049		hypothetical protein	1	1	38
group_5993		hypothetical protein	1	1	38
group_5972		hypothetical protein	1	1	38
group_5903		hypothetical protein	1	1	38
group_5889	sorA_2;	PTS system sorbose-specific EIIC component;hypothetical protein	1	1	38
group_5853		hypothetical protein	1	1	38
group_5739		hypothetical protein	1	1	38
group_5601		hypothetical protein	1	1	38
dnaJ_2	dnaJ_2	Chaperone protein DnaJ	2	1	37
group_5524		hypothetical protein	1	1	38
group_5449		hypothetical protein	1	1	38
ydaF	ydaF	Putative ribosomal N-acetyltransferase YdaF	1	1	38
group_5297		hypothetical protein	1	1	38
group_5234		hypothetical protein	1	1	38
group_5233	comGC	ComG operon protein 3	1	1	38

ahpF_ahpF_1	ahpF;ahpF_1	NADH dehydrogenase;Alkyl hydroperoxide reductase subunit F	2	0	38
group_5194	cutC	Copper homeostasis protein CutC	1	1	38
manR_1_birA_2	manR_1;birA_2;	Transcriptional regulator ManR;Bifunctional ligase/repressor BirA;hypothetical protein	1	1	38
rspR_1	rspR_1	HTH-type transcriptional repressor RspR	1	1	38
group_5039		hypothetical protein	1	1	38
group_5008		hypothetical protein	1	1	38
comEC_comEC_1	comEC;comEC_1	ComE operon protein 3	1	1	38
group_4950		hypothetical protein	1	1	38
chiD	chiD	Chitinase D	1	1	38
group_4668	yvoA_1	HTH-type transcriptional repressor YvoA	1	1	38
group_4610		hypothetical protein	1	1	38
group_4535		hypothetical protein	1	1	38
group_4430	agrB_1	Accessory gene regulator protein B	1	1	38
group_4305	nadR	Transcription repressor NadR	1	1	38
group_4246		hypothetical protein	1	1	38
group_4245		hypothetical protein	1	1	38
mapP_1	mapP_1	Maltose 6'-phosphate phosphatase	2	1	37
group_3865		hypothetical protein	1	1	38
group_3830		DegV domain-containing protein	1	1	38
group_3812		hypothetical protein	1	1	38
sorA_4	sorA_4	PTS system sorbose-specific EIIC component	1	1	38
group_3723		Hyaluronate lyase	1	1	38
ugl_1	ugl_1	Unsaturated chondroitin disaccharide hydrolase	1	1	38
group_3634		hypothetical protein	1	1	38
group_3526		hypothetical protein	3	13	22
group_3508		hypothetical protein	1	1	38
group_3420	manZ_2	PTS system mannose-specific EIID component	1	1	38

group_3041		hypothetical protein	1	1	38
group_3036	ytlR	Putative lipid kinase YtlR	1	1	38
esaA	esaA	ESAT-6 secretion accessory factor EsaA	1	2	37
group_3012		hypothetical protein	1	1	38
group_2579		hypothetical protein	1	1	38
group_2522		hypothetical protein	1	1	38
group_2495		hypothetical protein	1	1	38
group_2403	yqeN	putative protein YqeN	1	1	38
group_2321		putative ABC transporter permease	3	5	29
group_2231		hypothetical protein	1	1	38
group_1884		hypothetical protein	2	1	37
group_1505		hypothetical protein	1	1	38
manX_2	manX_2	PTS system mannose-specific EIIAB component	1	1	38
group_1385		hypothetical protein	1	1	38
group_1213		hypothetical protein	2	1	37
group_1169		hypothetical protein	1	1	38
gbpA_2	gbpA_2	GlcNAc-binding protein A	1	1	38
recD2	recD2	ATP-dependent RecD-like DNA helicase	1	1	38
comGA	comGA	ComG operon protein 1	1	1	38
group_784	bga	Beta-galactosidase	1	1	38
group_765		hypothetical protein	1	2	37
group_469		hypothetical protein	1	1	38
def1	def1	Peptide deformylase 1	1	1	38
group_68		hypothetical protein	1	2	37
manP_2	manP_2	PTS system mannose-specific EIIBCA component	1	1	37
group_7165		hypothetical protein	1	1	37
group_6688		hypothetical protein	3	4	33

group_6370		hypothetical protein	3	3	34
essB	essB	ESAT-6 secretion machinery protein EssB	1	2	36
group_5755		hypothetical protein	0	1	38
malP_2_malP_1	malP_2;malP_1	PTS system maltose-specific EIICB component	1	2	36
group_5719		hypothetical protein	1	1	37
group_5325		hypothetical protein	1	0	38
manP_3_manP_5	manP_3;manP_5	PTS system mannose-specific EIIBCA component	1	1	37
group_3627		hypothetical protein	1	1	37
group_3410		hypothetical protein	1	1	37
ywqN	ywqN	Putative NAD(P)H-dependent FMN-containing oxidoreductase YwqN	1	1	37
group_2379		18 kDa heat shock protein	1	0	38
group_1929		hypothetical protein	1	0	38
group_1621		putative phosphotransferase enzyme IIB component	1	1	37
esxA	esxA	ESAT-6 secretion system extracellular protein A	1	1	37
group_749	malL_3	Oligo-16-glucosidase	9	0	30
hepT	hepT	Heptaprenyl diphosphate synthase component 2	0	1	38
group_263		hypothetical protein	1	0	38
group_6669		hypothetical protein	2	0	36
essC_2_essC	essC_2;essC	ESAT-6 secretion machinery protein EssC	1	1	36
group_4622	ugpQ	Glycerophosphodiester phosphodiesterase cytoplasmic	1	0	36
yjcS_2	yjcS_2	Putative alkyl/aryl-sulfatase YjcS	2	0	36
group_3265		hypothetical protein	1	1	36
group_2748		hypothetical protein	1	0	37
group_323		hypothetical protein	1	1	36
folT_1	folT_1;	Folate transporter FolT;hypothetical protein	1	5	31
group_6019	relG	Toxin RelG	1	4	32
malH	malH	Maltose-6'-phosphate glucosidase MalH	1	2	34

glvR_1	glvR_1	HTH-type transcriptional regulator GlvR	1	2	34
group_3194		Pesticidal crystal protein Cry22Aa	1	1	35
group_2252		hypothetical protein	1	1	35
group_252		hypothetical protein	1	1	35
sorA_1	sorA_1	PTS system sorbose-specific EIIC component	1	4	30
manZ_1	manZ_1	PTS system mannose-specific EIID component	1	4	30
group_90		hypothetical protein	1	4	30
group_6163		hypothetical protein	1	2	32
group_6134		hypothetical protein	1	2	32
group_6051		hypothetical protein	1	2	32
group_4716		hypothetical protein	5	6	25
group_6181		hypothetical protein	1	4	29
group_5737		hypothetical protein	0	2	32
group_5359		hypothetical protein	1	2	32
ecfT_2	ecfT_2	Energy-coupling factor transporter transmembrane protein EcfT	1	4	29
ykoD	ykoD	Putative HMP/thiamine import ATP-binding protein YkoD	1	4	29
group_4930		hypothetical protein	2	0	31
group_4306		hypothetical protein	1	1	31
group_1726		hypothetical protein	1	1	31
group_503		hypothetical protein	1	1	31
group_6487	purQ	Phosphoribosylformylglycinamidine synthase subunit PurQ	2	0	30
group_6018		hypothetical protein	1	2	30
group_5852		hypothetical protein	1	1	29
rafB_rafB_1	rafB;rafB_1	Raffinose permease	1	1	29
group_2759		hypothetical protein	1	1	29
group_2749		Extracellular exo-alpha-(1->5)-L-arabinofuranosidase	1	1	29
group_2409		hypothetical protein	1	1	29

group_2177		hypothetical protein	1	1	29
group_795		hypothetical protein	1	1	29
group_7944		hypothetical protein	1	1	30
group_7166		hypothetical protein	5	3	23
group_5471		hypothetical protein	1	0	31
group_677		hypothetical protein	1	0	31
group_6972		hypothetical protein	1	1	29
group_4807	hrtA_2	Putative hemin import ATP-binding protein HrtA	0	3	25
group_2310		putative ABC transporter permease	0	3	25
group_1402		hypothetical protein	0	3	25
group_138		hypothetical protein	1	1	27
group_5463		hypothetical protein	1	0	28
xerC_3__IntTn_xerD_1_xerC_1	xerC_3;;Int-Tn;xerD_1;xerC_1	Tyrosine recombinase XerC;Putative prophage phiRv2 integrase;hypothetical protein;Transposase from transposon Tn916;Tyrosine recombinase XerD	0	4	21
group_3876		hypothetical protein	0	0	26
rppH	rppH	RNA pyrophosphohydrolase	0	0	24
group_5760		hypothetical protein	1	1	22
yknY_2_yxdL_1	yknY_2;yxdL_1	putative ABC transporter ATP-binding protein YknY;ABC transporter ATP-binding protein YxdL	1	0	25
group_4280		hypothetical protein	0	0	24
group_2407		hypothetical protein	0	1	25
group_2176		hypothetical protein	0	1	25
group_8179		hypothetical protein	1	0	24
group_6964		hypothetical protein	0	0	20
